# Restriction of the Global IgM Repertoire in Antiphospholipid Syndrome

**DOI:** 10.3389/fimmu.2022.865232

**Published:** 2022-04-13

**Authors:** Shina Pashova, Lubomir Balabanski, Gabriel Elmadjian, Alexey Savov, Elena Stoyanova, Velizar Shivarov, Peter Petrov, Anastas Pashov

**Affiliations:** ^1^Institute of Biology and Immunology of Reproduction, Bulgarian Academy of Sciences, Sofia, Bulgaria; ^2^Department of Medical Genetics, Medical University-Sofia, Sofia, Bulgaria; ^3^Genomics Laboratory, Hospital “Malinov”, Sofia, Bulgaria; ^4^ICON plc, Sofia, Bulgaria; ^5^Institute Mathematics and Informatics, Bulgarian Academy of Sciences, Sofia, Bulgaria; ^6^Institute of Microbiology, Bulgarian Academy of Sciences, Sofia, Bulgaria

**Keywords:** IgM, antibody repertoire, antiphospholipid syndrome, Igome, deep panning, sequence graphs

## Abstract

The typical anti-phospholipid antibodies (APLA) in the anti-phospholipid syndrome (APS) are reactive with the phospholipid-binding protein β2GPI as well as a growing list of other protein targets. The relation of APLA to natural antibodies and the fuzzy set of autoantigens involved provoked us to study the changes in the IgM repertoire in APS. To this end, peptides selected by serum IgM from a 7-residue linear peptide phage display library (PDL) were deep sequenced. The analysis was aided by a novel formal representation of the Igome (the mimotope set reflecting the IgM specificities) in the form of a sequence graph. The study involved women with APLA and habitual abortions (n=24) compared to age-matched clinically healthy pregnant women (n=20). Their pooled Igomes (297 028 mimotope sequences) were compared also to the global public repertoire Igome of pooled donor plasma IgM (n=2 796 484) and a set of 7-mer sequences found in the J regions of human immunoglobulins (n=4 433 252). The pooled Igome was represented as a graph connecting the sequences as similar as the mimotopes of the same monoclonal antibody. The criterion was based on previously published data. In the resulting graph, identifiable clusters of vertices were considered related to the footprints of overlapping antibody cross-reactivities. A subgraph based on the clusters with a significant differential expression of APS patients’ mimotopes contained predominantly specificities underrepresented in APS. The differentially expressed IgM footprints showed also an increased cross-reactivity with immunoglobulin J regions. The specificities underexpressed in APS had a higher correlation with public specificities than those overexpressed. The APS associated specificities were strongly related also to the human peptidome with 1 072 mimotope sequences found in 7 519 human proteins. These regions were characterized by low complexity. Thus, the IgM repertoire of the APS patients was found to be characterized by a significant reduction of certain public specificities found in the healthy controls with targets representing low complexity linear self-epitopes homologous to human antibody J regions.

## 1 Introduction

A persistently elevated level of antiphospholipid antibodies (APLA) is a sign of anti-phospholipid syndrome (APS). Clinical manifestation of APS depends on triggers like infections or pregnancy and causes pregnancy complications, thrombosis, or neurological symptoms (1, 2). Originally discovered as autoantibodies to the membrane lipid cardiolipin (3) and cross-reactive to other membrane phospholipids (4), they were suggested to be related to the natural antibodies that serve in the process of clearance of apoptotic cells (5, 6). Most of the pathogenic APLA are specific not to a phospholipid but to the PLB protein β2GPI (7, 8). Further, other protein targets of APLA - prothrombin, annexin A5, protein C (9–11), have also been found. The increasing number of discovered APLA targets prove that this autoreactive group of antibodies is heterogeneous (12) and suggest that more, still unknown, targets exist. APLA are suggested to activate factors of the coagulation system or to decrease the anticoagulation capacity of Annexin A5 and Protein C (13, 14). A key target of APLA seems to be also the phospholipid-bound EPCR (Epidermal Protein C Receptor). Binding to it triggers coagulation and an autoimmune circuit for expansion of APLA secreting B1a cells in a mouse model (15). The relation of APLA to natural antibodies and the finding of a dysregulated B1a cell stimulation in mice warrant the deeper study of the changes in the human IgM repertoire in APS to explore potential new mechanistic evidence and diagnostic opportunities.

The natural IgM repertoire is selected to be moderately self-reactive (16, 17). Despite the immense sequence diversity, part of it is highly reproducible across individuals (public specificities) (18–20) and organized in clusters which can be projected as an organized set of mimotopes (21). As part of the housekeeping functions of IgM, this compartment serves as a nonredundant enhancer of B cell tolerance (22, 23). The absence of polyclonal secretory IgM or of its B cell restricted receptor - FcµR, impacts the early repertoire selection and tolerance induction of B2 and B1 populations in mice and leads to the development of systemic antibody autoreactivity (23–26). Thus, repertoire-wide analysis of the global reactivity of this important compartment is promising to reveal useful knowledge about pronounced/pernicious autoimmunity.

The natural autoantibodies are engaged in idiotypic interactions (27, 28) and that also applies to the non-pathogenic cofactor-independent antiphospholipid antibodies (29–32).

With these considerations, it is conceivable that a panel of particular APLA may fall short as a probe of the antibody repertoire changes in APS. Global repertoire view contains disease-related signatures (33–35) and the recurrent public specificities can be a source of diagnostic profiles (21) shaped by diverse mechanisms. Therefore, we set out to study IgM antibody changes in APS patients using a repertoire level functional assay (Igome (36)). Applying a novel formal representation of the Igome, here we report the findings of some counterintuitive changes in the IgM repertoire which could be explained by selection-dependent idiotypic relations (37).

## 2 Materials and Methods

### 2.1 Serum Samples

Patients were recruited at two sites – The National Genetics Laboratory at The University Clinic for Obstetrics and Gynecology in Sofia and at Repro Inova Lab, Sofia. Serum samples (0.5 ml) from 24 healthy pregnant women in the first trimester and from 20 APLA positive women (diagnosed with APS) with spontaneous abortions were collected after informed consent. Healthy pregnant women were selected as the suitable control since the APLS patients were typically diagnosed after an abortion and the antibody repertoire dynamics are supposed to be in the timescale of weeks. Thus, the repertoire status of women with a recent abortion might be closest to that of women with a similar stage of the pregnancy. The collection and the following studies were approved by the Human Studies Ethics Committee of the Institute of Biology and Immunology of Reproduction at the Bulgarian Academy of Sciences. The samples were anonymized and actual coding of the specimens was not necessary as the immunoglobulin fractions were mixed in two pools. The samples were kept frozen at -15°C until processing.

### 2.2 IgM Isolation

Each serum sample was thawed and incubated at 37°C for 30 min for the dissolution of IgM complexes. Next, the sera were centrifuged and 30-fold diluted with PBS followed by ultrafiltration (Amicon-Ultra 100kDa membranes, Millipore) for initial fractionation of serum proteins above 100 kDa. The high-molecular fraction of serum proteins was applied in a series on columns (HiTrap Protein G High Performance and HiTrap IgM Purification, GE Healthcare) for affinity purification of IgM according to the manufacturer’s instructions. The IgM fractions from each serum sample were mixed in 2 pools: 1 – IgM from healthy pregnant women, 2 - IgM from women with APS.

### 2.3 Deep Panning

The deep panning technique used was previously described (36, 38, 39). The immunoglobulin fractions were diluted to 0.1 mg/ml in coating buffer and ON adsorbed on polystyrene flasks. Following a blocking step, 10mcl of a 7-mer random peptide library (E8100S, Ph.D.-7, New England Biolabs, Ipswitch, MA) were appropriately diluted and panned on the adsorbed immunoglobulins according to the protocol of the manufacturer. Non-binding phages were strictly washed-away while binding phages were eluted with glycine buffer at pH 2.7 and immediately brought to pH 7. The eluate was then adsorbed on purified monoclonal IgM from myeloma to subtract phages binding to constant antibody regions (negative selection). The non-binding fraction from the monoclonal IgM adsorption step was collected and amplified once in E. coli (strain K12 ER2738).

The amplified phages were precipitated and purified from the culture media according to the manufacturer’s instructions. M13 phage genomic DNA was isolated using Qiagen plasmid kit (Qiagen, Hilden, Geramany) with Qiagen supplementary protocol for isolation of single-stranded DNA from M13 phage. The isolated DNA was subjected to PCR using Q5 High-Fidelity PCR Kit (New England Biolabs, Ipswitch, MA) for amplification of the 21-base library inserts. The primers, flanking the library insert and the reaction conditions were after Matochko et al., 2012 (38).

The 63 bp product (including insert and primers) from the PCR amplification was precipitated with 70% ethanol/300mM sodium acetate/20mM MgCl_2_ ON, -20°C, washed with ethanol several times and air-dried. The pellet was solubilized in water and applied on 3% agarose gel for electrophoresis at 140 V for 1 hour. Bands were visualized with EtBr. The band corresponding to 63 bp (according to DNA ladder – New England Biolabs, Ipswitch, MA) was excised and the PCR fragments were extracted from the gel using QIAEX II Gel Extraction Kit (Qiagen, USA). Concentration and quality checks were conducted using capillary electrophoresis. 63 bp PCR fragments from each group were blunt-end repaired, 3’ adenylated, ligated to Illumina adapters, and enriched through PCR amplification (PCR Primer PE 1.0 and 2.0) using Illumina Paired-End Sample Prep Kit (Illumina, USA) and according to the provided kit protocol. The sequencing was performed on a MiSeq System (Illumina, USA).

### 2.4 Data Processing

#### 2.4.1 Preparation and Cleaning of the Sequence Libraries

The fastq files from the sequencing step were processed using the algorithm proposed by (38) using high accuracy (Q score > 32). This step yielded quality filtered unique peptide sequences and their copy number. All further data processing was done using the R software environment (packages *igraph, stringdist, pROC, parallel, stringi, Biostrings, Rcapture, qualV*, *pcor*, *entropy*, etc.). Further, the libraries were labeled and pooled together with ones from previous IgM public Igome analyses (21) to determine the frequency of occurrence of each sequence. This was done to expand the range of sequences that meet the criteria of copy number between 3 and 10 (21). Thus, erroneous sequences and sequences from overgrowing phage clones were avoided as possible. Next, the SAROTUP (40) algorithm was used to detect potential artifactual target unrelated sequences which were discarded.

#### 2.4.2 Selecting a String Metric Suitable for Mimotope Comparisons

To study the typical sequence relations between mimotopes of the same monoclonal antibody, 49 mimotope sets from previously published panning experiments with monoclonals and the same phage display library were downloaded from the Biopanning Data Bank (http://i.uestc.edu.cn/bdb/) (41). The different string metric functions were available from the R package *stringdist* and the pairwise alignment function was from Biostrings. The biophysical property based similarity of amino acids expansion of the string metrics was achieved by recoding the amino acids according to their properties grouping them in the following 15 partially overlapping groups: M - M,I,L,V; F - F,W,Y, Y - Y,H; D - D,E; N - D,N; H - H,N; S - S,N; E - E,Q; K - E,K; R - R,K,Q; A - S,A; T - T,S; G - G; C - C; P – P. The groups were formed based on pairs with positive substitution scores in the BLOSUM2 table. A residue was considered equivalent to another if they belonged to the same group irrespective of the fact that they could participate in other groups too. In this way, the string editing metrics could be applied in combination with the residues’ approximate chemical and evolutionary equivalence. These extended amino acid sequence similarity metrics were compared to the original editing distances applied (which consider each amino acid residue unique).

To compare the different metrics, ROC curves were constructed based on isospecific and randomly paired mimotopes. The editing distances between pairs of mimotopes belonging to the same monoclonal group were compared to the distance between pairs of sequences representing a mimotope from the monoclonal groups and a sequence sampled from our public mimotope library as a random mimotope reference. The AUC and the cutoff parameters were bootstrapped over 20 random samples of public mimotopes.

#### 2.4.3 The Graph

Having selected LCS (longest common subsequence) distance as the most appropriate metric to detect specificity relation between mimotopes next the pooled library of mimotopes of IgM from control and APS patients was represented as a graph. To this end, the sequences adjacent to each sequence were found using as a criterion for linkage LCS distance of less than 5. This means edges were drawn between vertices (sequences) which shared a longest common subsequence of 5 or 6 letters (one sequence can be transformed into the other by 4 or fewer insertions/deletions). Thus, an adjacency list was constructed which was melted into an edge list and the latter was used to build the graph using the *igraph* package. The calculation of the adjacency lists was done on the Avitohol HPC cluster at the High-Performance Computing Center of the Bulgarian Academy of Sciences. The comparison to other libraries (public mimotope library and the library of putative idiotopes) was achieved by finding neighbors from those libraries to the vertices of the studied graph (n=3.9x10^5^). This reduced drastically the computation time as compared to building a giant graph encompassing all available sequences (n=8x10^6^).

The degree, eigencentrality, transitivity, etc. graph parameters were calculated using the *igraph* package. The Gephi software (42) was used for the visualization of the graphs by exporting the graph from R to Gephi using the graphml format. All vertex properties of interest (partition by diagnosis, number of neighbors from other libraries, etc.) were exported as appropriately scaled vertex attributes.

#### 2.4.4 Clustering on the Graph

Most of the graph clustering algorithms are so computationally demanding that they are applicable only to small graphs. The graph of the mimotopes proved extremely connected so the fast Louvain algorithms (43) was chosen. A recursive clustering function based on the Louvain clustering function (44) was implemented yielding a tree structure. The ultimate clustering was obtained by cutting the tree at different levels and choosing the level which maximizes the number of clusters of more than 3 vertices. The quality of the clustering was quantitated using the modularity function.

#### 2.4.5 Searching the Proteome and the Linear Epitope Database

To use the clusters defined as patterns for searching sequence databases the simplest model of position-specific scoring matrices (PSSM) was used. It was shown to differentiate with very high sensitivity (0.97) and specificity (0.98) the sequences belonging to the cluster from random pairs. The linear B cell epitopes were accessed at the Immune Epitope Database [http://www.iedb.org/, (45)] and 136 884 sequences longer than 6 letters were recovered with their annotation. Each cluster model in the form of PSSM was used to search the epitopes with a sliding window when they were longer than 7 residues by summing up the position specific scores (negative logarithm of the probability of the amino acid residue at the given position) for each frame. Sequences containing a frame scoring better than the threshold value for the model were considered homologous to the cluster.

The collection of the proteome sequences was accessed through UniProt (https://www.uniprot.org/). Using the PSSM scoring to find positive hits in the proteome (effectively – the peptidome, because of the sliding frame interrogating each possible short sequence) lead to over 25 000 hits. To make the search more stringent only exact matches to any of the peptides were considered. This approach produced 7 519 hits.

The entropy of the sequences both of the mimotopes and from the peptidome was calculated using the R package *entropy* and the bias-corrected maximum likelihood method.

#### 2.4.6 Statistical Methods

The number of neighbors of a single vertex or the cluster **A/C** partition were compared using proportion test for more than 30 elements and binomial test for the smaller counts. Partitions of counts in multiple groups were assessed using the Chi-square test. Regressions were analyzed by linear regression models. Distributions of mean degree, eigencentrality or mean neighbor counts were bootstrapped to estimate quantile ranges. All tests on vertex degree, eigen centrality, transitivity, number of neighbors, except for the Chi-square or proportion tests were carried on log-transformed data to stabilize the variance.

#### 2.4.7 Data Availability

All code and data are available at https://github.com/ansts/aPLAb1.

## 3 Results

### 3.1 Mimotope Libraries Generation

Mimotope libraries corresponding to the IgM repertoires of healthy pregnant women (Control – **C)** and women with APLA (**A**) diagnosed after a spontaneous abortion were used to study changes in the global antibody repertoire in APS. They were generated by deep panning of 7-mer PDL (Ph.D.-7, NEB), with an expected diversity of 10^9^. The PDL was selected on pools of affinity-purified total IgM from control (n = 24) and APLA positive women (n = 20). Library **C** contained 109 617 and library **A** - 297 028 unique mimotopes. Thus, the combined library (**C**+**A**) contained 391 422 unique mimotopes (15 223 were common). In addition, a comparison was made to 2 796 484 mimotopes of global public reactivities from a repeated previous experiment (21) with IgM from 10 000 healthy donors (library **G)**. Also, a set of 217 442 7-mer sequences from a previously published (46, 47) analysis of the same PDL without prior selection was used as a background non-selected library **B**.

Library **B** was deep sequenced following the same protocol so library **B** could be considered a random sample from the PDL. Consequently, the intersection of **B** with our pooled libraries (**A**+**C**+**G**) could be used to infer the actual diversity of the sampled 7-mer sequence space borrowing the capture-recapture approach from ecology (48). The estimate of the initial diversity of PDL was 0.88x10^8^ (5-95%: 0.86-0.9 x10^8^). In comparison, the intersection with a sample of random peptides of the same size as **B** but with a uniform residue frequency yielded an estimate of the sampling space at 12.8 x10^8^ (5-95%: 11.9-13.7 x10^8^) corresponding to the expected full 7-mer diversity of 20^7^.

**Figure 1:**
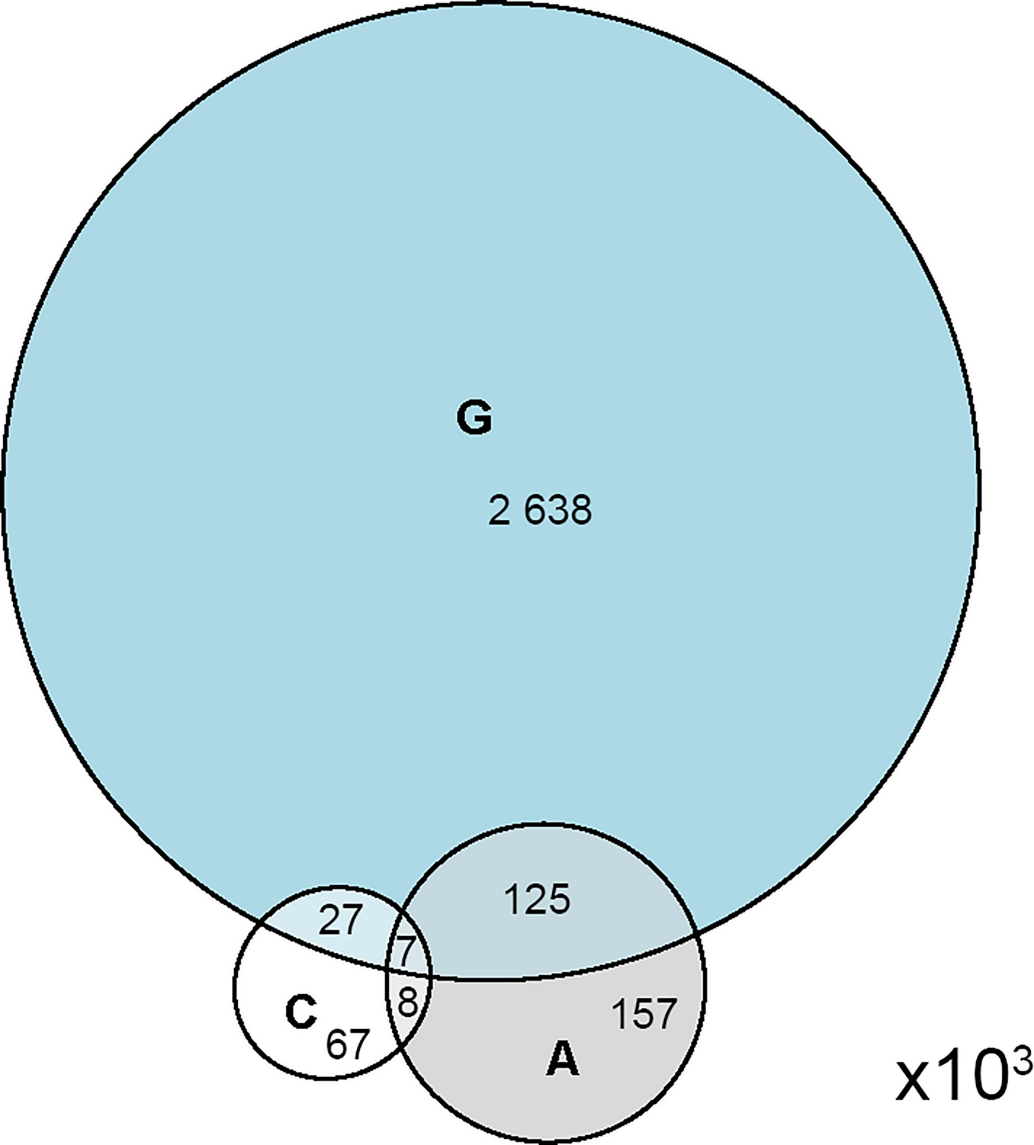
Venn diagram representing the relative size and overlap between the three deep panning selected mimotope libraries.

The estimated original sequence space allowed for sizing the significance of the overlap between the selected libraries (**Figure 1**). All pairs of selected libraries (**A/C**; **A/G** and **C/G**) had significantly larger overlaps than predicted by random sampling from 0.88x10^8^ sequences (proportion test, p<0.001). The overlaps for **A** and **C** with **G** library were respectively 9.6 and 13.9-fold, and between **A** and **C** - 41.2-fold higher than expected by chance. Thus, **C** and **A** libraries were more similar than any of them to the public repertoire indicating that the studied groups together represent a subset with its own public specificities. This was considered also an evidence of appropriately matched control subjects.

### 3.2 Formal Representation of the Igome as a Map of the IgM Repertoire

**Figure 2:**
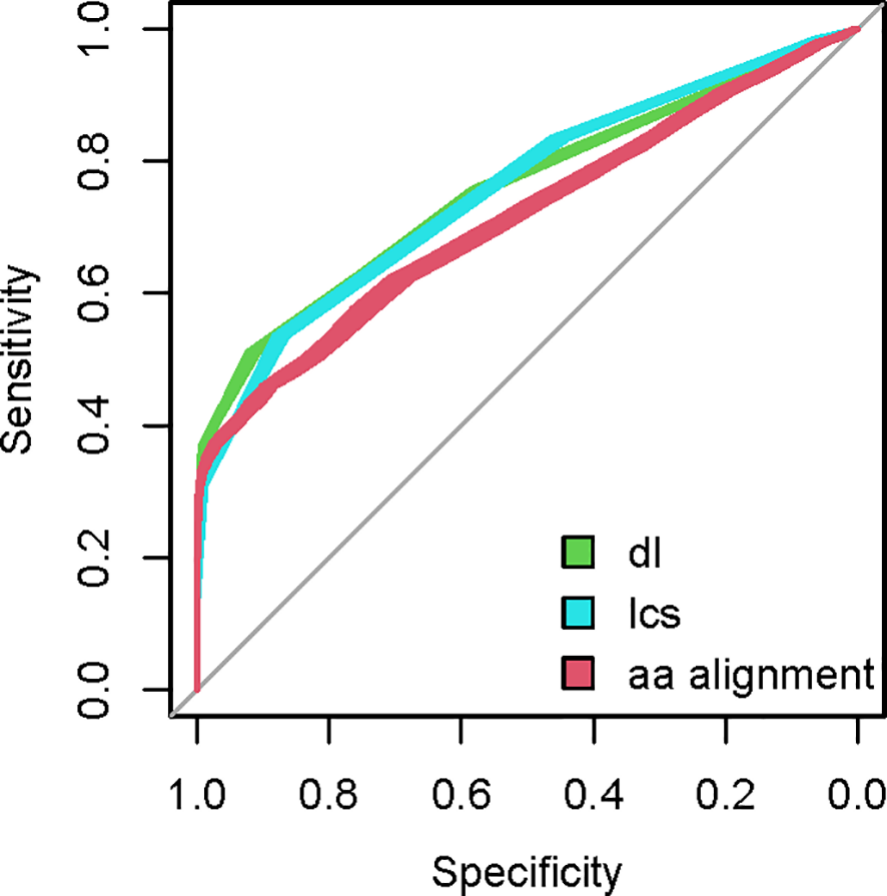
Examples of ROC curve sets for three string metrics - Demerau-Levenstein (dl), longest common subsequence distance (lcs) and pairwise alignment based on the BLOSUM62 scoring matrix. Twenty ROC curves are constructed with each metric using 20 random samples of G library sequences to pair with the mimotopes of known mAb from the Biopanning Data Bank and compared to isospecific pairs from the data bank.

**Figure 3:**
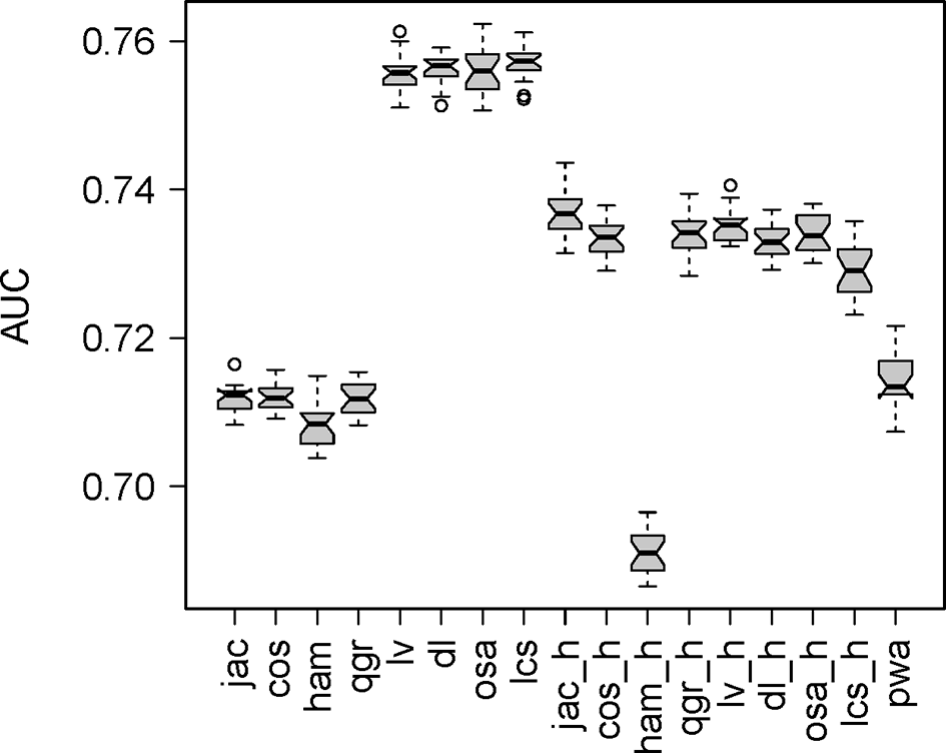
Box plot of the area under the ROC curve for the detection of isospecific pairs of mimotopes using diverse string metrics. Ham - Hamming distance, lcs - longest common subsequence distance, lv - Levenshtein distance, dl - Damerau-Levenshtein distance, osa - optimal string alignment, qgr - q-gram distance, cos - cosine distance between q-gram profiles, jac - Jaccard distance between q-gram profiles, pwa - pair-wise alignment (BLOSUM62 substitution matrix based), …_h - versions of string distances with recoded amino acids (AA) based on the Phys\Chem properties. The recoding of the AA aimed at assigning the same letter to AA of the same property but when one AA was grouped in more than one property set the letter was chosen at the point of comparison to match (if possible) the property set of the counterpart AA. This approach was meant to make possible the use of string distance metrics in the context of physicochemical properties matching.

A set of mimotopes selected by an entire repertoire (Igome) can be used as a tool to study the selecting repertoire. The Igome is a convolution of overlapping footprints of cross-reactive antibody clones. Our data cannot match particular clones to mimotope subsets so these footprints had to be inferred at least approximately. To do this, sequence (if not structural) similarities between isospecific (to the same monoclonal - mAb) mimotopes measured by string metrics or pairwise alignment could be calibrated on existing data. Previously published sets of mimotopes of known mAbs are available from the Biopanning Data Bank (41). After filtering for mimotopes derived by panning the same PDL, 337 mimotopes of 49 mAb were selected. The mimotope groups ranged from 2 to 27 sequences. Pairs of isospecific mimotopes were used as a positive data set (**Supplementary Data Sheet 1**). They were compared to a set where each of these sequences was paired to a random mimotope from **G** library. To compare different string metrics, ROC curves were constructed plotting the specificity/sensitivity tradeoffs at different cut-off values. The mean area under the curve (AUC) for different metrics was bootstrapped using 20 samples of irrelevant peptides from **G** (**Figure 2**). The AUC comparison indicated the editing distances excelled in detecting isospecific mimotopes (**Figure 3**). The longest common subsequences (LCS) distance was selected as computationally most efficient. This metric counts the number of deletions and insertions necessary to convert one of a pair of sequences into the other (see **Supplementary Figure 1** for an example).

**Figure 4:**
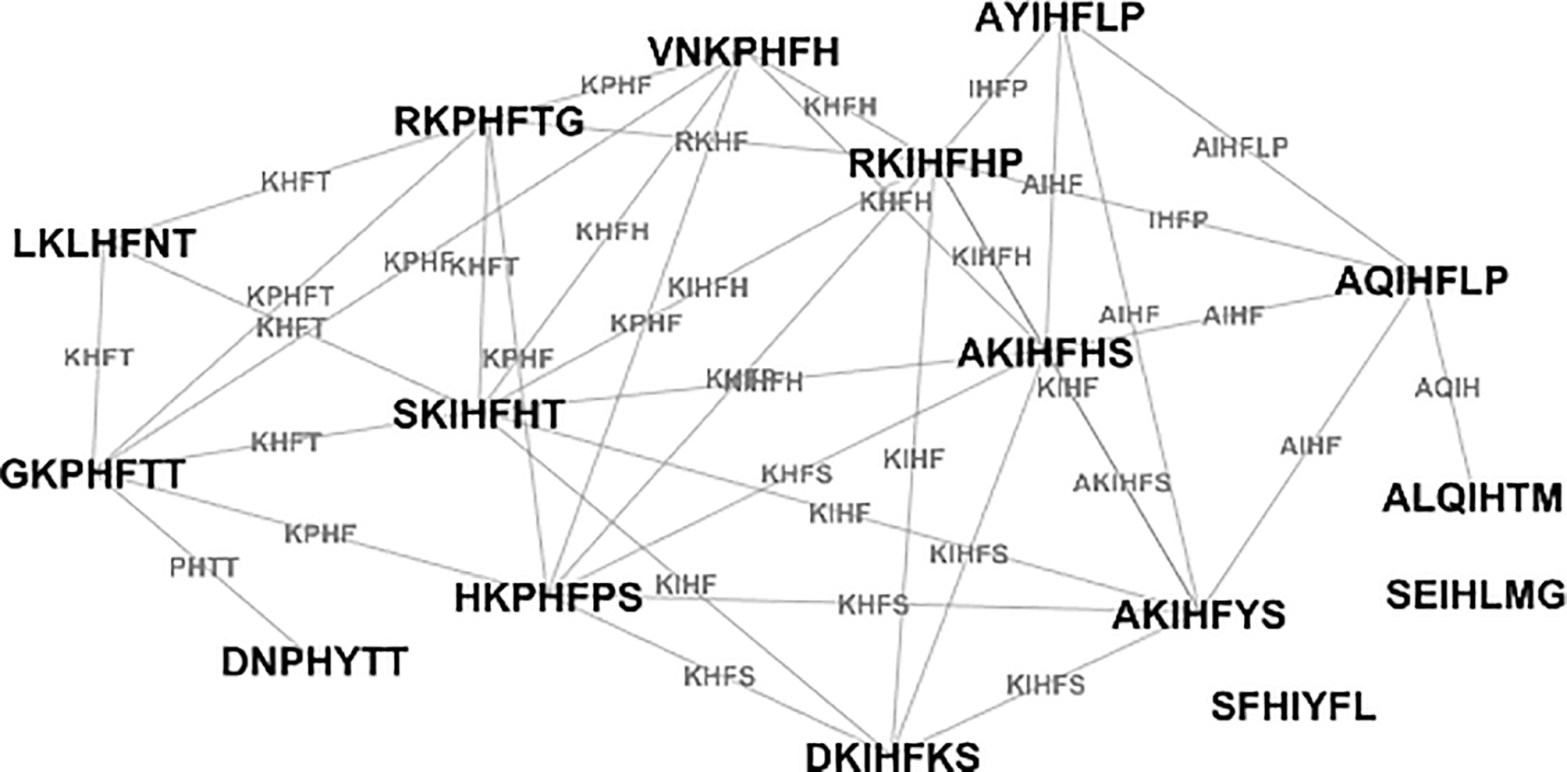
A typical graph of the sequences of mimotopes of a single monoclonal – example based on the SA-7 anti-Fas antibody (49). The edge names are the respective longest common subsequences.

Having a suitable metric to measure isospecific mimotope similarities allowed for building a graph of the IgM Igome in APS. A similar approach was used by Miho et al. (20) for repertoire sequencing data. To allow for analysis of the differential expression of reactivities, the graph pooled **A** and **C** libraries. The vertices of the graph (mimotope sequences) were connected when their distance implied up to 4 deletions/insertions or LCS of at least 5 letters common for both sequences. The ROC curve analysis suggested optimal threshold was LCS of 4 letters but this criterion had low specificity. Increasing the criterion by one common residue provided specificity > 0.9, focusing on the isospecific pairs in a stricter sense (**Figure 4**).

**Figure 5:**
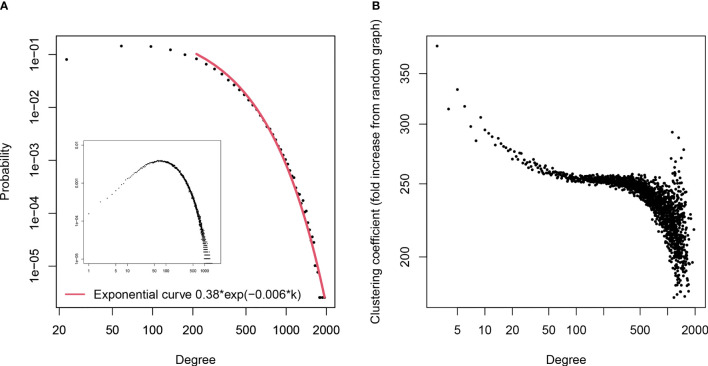
**(A)** - Distribution of the degrees in the graph of the pooled mimotopes from libraries A and C The distribution was plotted using bins splitting the degree range in 50 equal ranges (linear bins) rather than the discrete degree values. The distribution is exponential (R^2^ = 0.99, p<10^-7^). The distribution plot based on the degree values without binning is shown in the inset. This type of degree distribution is consistent with a network evolving in time by random (non-preferential) attachment which can be a sign of positive selection of the IgM repertoire of specificities. **(B)** – dependence of local clustering coefficient on degree. The clustering coefficient decreasing with degree and much higher than the expected level of mean(degree)/size of network is a confirmation that the graph is not randomly connected but an evolving network with the newer specificities related to the older ones.

The undirected graph, thus constructed, had 391 396 vertices and 41 263 592 edges. Twenty-six sequences were disregarded as singlets. The graph was highly connected. The graph diameter was 11 with 99.3% of the paths shorter than 7 edges which is a typical small-world network. The exponential distribution of degrees as well as the high and degree-dependent local clustering coefficient (**Figure 5**) indicated an evolving network with newer clones always cross-reactive with at least one older clone picked randomly (50). Overall, the graph corresponds to a dense network of polyspecific clones selected to be cross-reactive. In addition, the graph exhibited a very high degree assortativity – 0.51, indicating the existence of high- and low-density footprints.

### 3.3 Mapping Differentially Expressed IgM Specificities

#### 3.3.1 Neighborhoods

**Figure 6:**
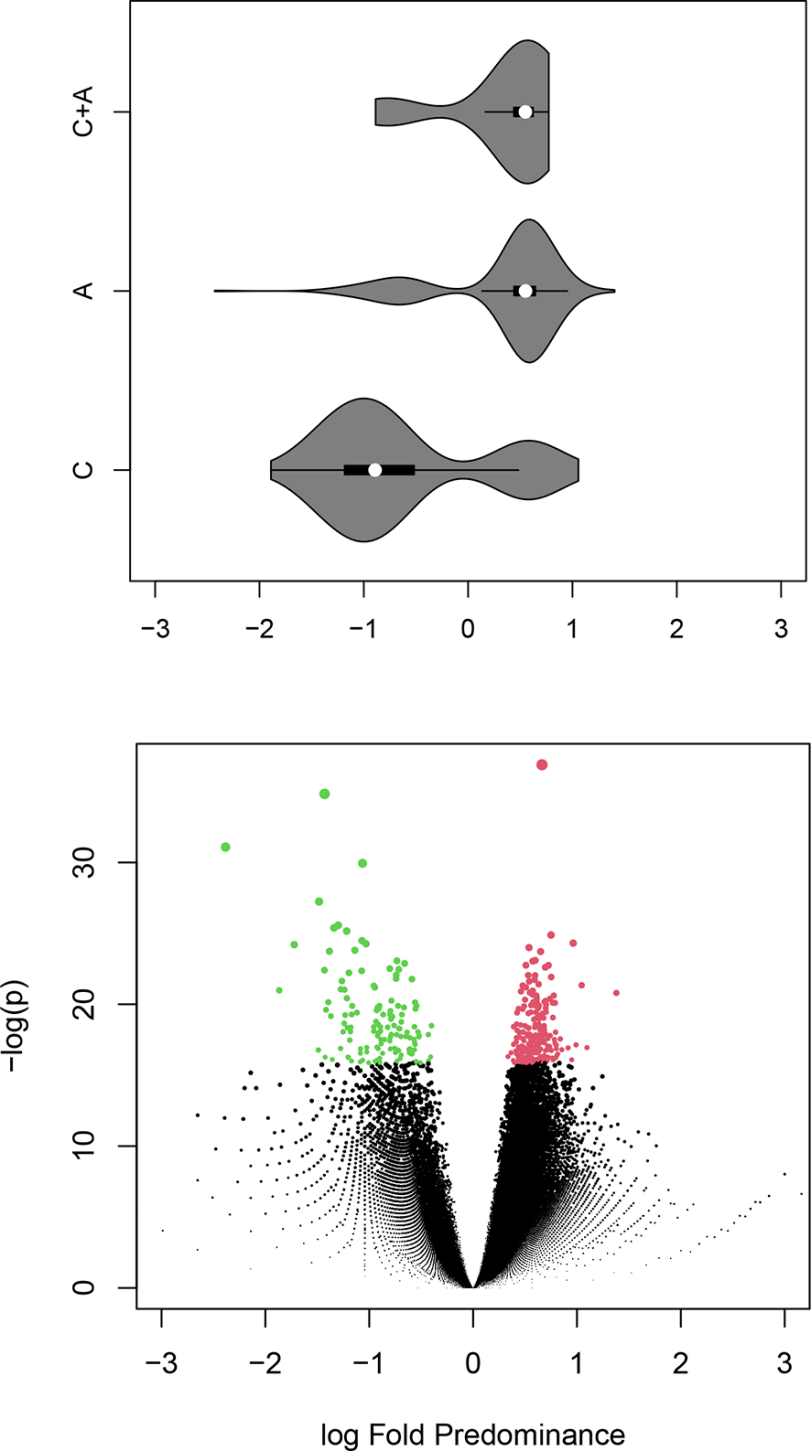
Bellow – volcano plot illustrating the differential representation of control or APS specific mimotope sequences in each (overlapping) neighborhood of the graph. After a Benjamini-Hochberg false discovery rate correction the significant 358 neighborhoods (red – **A** or green - **C**) contained 38% with predominance of **C** sequences while the percentage of **C** sequences in the library is 26%. Above – a violin plot corresponding to the fold difference on the X axis of the volcano plot showing the distribution of the central sequence for each neighborhood by group. The colors indicate the type of neighborhood. APS – anti-phospholipid syndrome.

Starting with the proposition that the Igome footprints of individual clones are represented by sets of related sequences, it was hypothesized that the neighborhood (connected sequences) of each vertex can be viewed as the smallest continuous fragment of such a footprint. Next, the proportions of each neighborhood’s **C** and **A** mimotopes were compared. The counts of the common sequences in each neighborhood were split and added to those of **C** and **A** equally (to err on the safe side). The distribution of the neighborhoods’ **A/C** proportions is illustrated in the volcano plot in **Figure 6**. The significant **C** neighborhoods were 38.3% while the frequency of **C** sequences was 26.1%. It was also more common to find **C** sequences in **A** neighborhoods than vice versa (**Figure 6**, violin plot). The sizes of the neighborhoods showed a considerable shift to higher numbers for the significant ones (**Supplementary Figure 2**) characteristic especially of the **A** neighborhoods (**Supplementary Figure 3**).

Thus, footprints of IgM specificities were found that were both overrepresented or underrepresented in APS patients. The APS overrepresented specificities were not exclusively specific for APS but rather marked a quantitative shift to lower diversity in a compartment of the repertoire of IgM specificities.

Next, the public specificities (**G**) were mapped to the **A+C** graph by counting of **G** neighbors to each vertex. In the differentially expressed neighborhoods, the numbers of **G** mimotopes correlated with the tendency of the specificities to be lost in APS (**Supplementary Figure 4**). Thus, APS IgM repertoire appeared to characteristically lose some public IgM specificities.

#### 3.3.2 The Significant Neighborhoods Induced Subgraph

**Figure 7:**
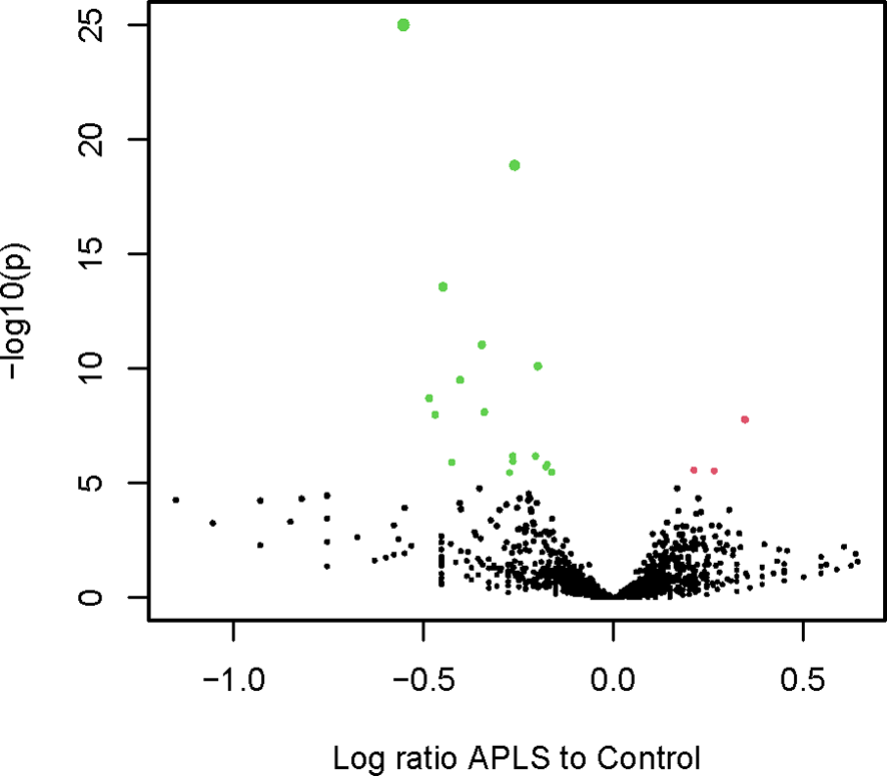
Volcano plot illustrating the differential representation of control or APS specific mimotope sequences in each cluster of sequences in the significant neighborhood graph (red – **A** or green - **C**).

A subgraph induced by a subset of a graph’s vertices is a part of that graph containing only that subset of vertices and all the edges existing between them in the original graph. To simplify the convoluted neighborhood set, an induced subgraph was extracted using the vertices included in the 358 significant neighborhoods (NG). The graph contained 70 796 vertices (15 323 C, 52 410 A, and 3063 found in both) and 7 853 656 edges. The mimotope sequences included in NG were further clustered using a recurrent Louvain clustering algorithm (43, 44). The clustering yielded 370 clusters ranging from 4 to 1546 sequences. The clusters smaller than 4 vertices were disregarded. Proportion test with Benjamini-Hochberg false discovery rate correction yielded only 4 clusters (sizes of 256 to 720) with a predominance of **A** mimotopes and 28 clusters (sizes of 10 to 1546, 19/28 were less than 256) with a predominance of **C** mimotopes (**Figure 7**). Thus, the non-overlapping cross-reactivity groups in APS showed a hole in the repertoire.

#### 3.3.3 The Global Graph Clusters

The neighborhoods focused on a formalism for antibody footprints in the Igome. On the other hand, the statistical test used to select differentially expressed ones filters out vertices with low degree which could skew the analysis to the most abundant IgM reactivities (e.g. – highly represented antibodies like anti-alpha-Gal, anti-phosphatidyl choline, blood group antigens, etc.). To get a more comprehensive view of the Igome the same recursive Louvain clustering algorithm was applied to the whole graph. It yielded 3585 workable clusters ranging from 4 to 1040 sequences. Proportion test with Benjamini-Hochberg false discovery rate correction yielded only 3 (sizes of 409 to 631) overrepresented and 17 clusters (sizes of 95 to 942, 11/17 were less than 409) underrepresented in **A** (**Supplementary Figure 5**), a result in agreement with the NG partition.

The two approaches produced different clusters. The overlap between **C** clusters from the NG clustering (NGcl) and the whole graph clustering (WGcl) was less than 6% for all but 2 pairs of clusters which had respectively 12% and 20% overlap. For the **A** clusters, this overlap was less than 7%. Thus, the two schemes of clustering yielded overall very different clusters with the same properties – **A** clusters were less in number (proportion test, p=0.0018 for WGcl and p=0.0005 for NGcl) and bigger (Wilcoxon rank-sum exact test, p=0.0397 for all clusters) than **C** clusters. The clusters were analyzed in parallel also further to ensure the consistency of the results for those two regions of the graph.

The **A** clusters in WGcl had a higher mean number of neighbors and a higher eigencentrality (high connectedness) than **C** clusters (**Supplementary Figure 6**). This effect was lost in the NGcl probably due to the higher density of the graph. Finally, the **A** clusters had a higher modularity than the **C** clusters and the average random set of clusters (**Supplementary Figure 7**). Together, these findings reinforce the hypothesis that the APS repertoire changes lead to an overexpression of a few IgM reactivities. The underexpressed cross-reactivities are a higher number and more overlapping especially in the dense part of the graph (NGcl).

#### 3.3.4 Mapping of Public Repertoire Specificities and Idiotopes to the APS IgM Igome Graph

As noted above (**Supplementary Figure 4**), the predominance of **C** in a neighborhood seemed to correlate strongly with the abundance of public **G** IgM mimotopes. On the other hand, the mean degree, mean eigencentrality, and the mean number of **G** neighbors seemed to change in parallel in NGc versus WGcl sets (**Supplementary Figure 6**). The underlying common cause seemed to be the higher density of the graph regions represented in NG. The topology of the graph is dependent on the distribution of the amino acid residues in the PDL. This distribution proved skewed ( (21) as well as here – in 3.1). Mapping the non-selected library **B** sequences to the graph neighborhoods yielded a distribution of **B** neighbor counts which correlated strongly with the degree of the vertices (R^2 =^ 0.77, p<1e-7). Thus, changes in any parameter of the vertices had to be controlled for this underlying dependence of degree on the PDL residue bias. A significant partial correlation between the number of **G** neighbors and the vertex degree remained (partial R^2^ = 0.77, p<1e-7) controlling for **B** indicating that in small pools of IgM repertoires (like **A** and **C**) the abundance of mimotopes for individual specificities depends on their relation to the public repertoire (**Supplementary Data Sheet 2**).

Previously, we found a significant homology between public mimotopes and short sequences found in immunoglobulin J regions (21). This was interpreted as a reflection of possible idiotype relations in the repertoire. The J regions homologous mimotopes could be considered mimics of (parts of) potential idiotopes. Consequently, it was tempting to retest conclusions of previous reports relating autoimmunity to idiotypes (29–32). Projecting the potential idiotopes onto the Igome graph and the **A/C** relations could help reconcile this contentious topic. Using the Identical Protein Groups database of NCBI, 4 433 252 unique 7-mer sequences were extracted from 1 024 389 human immunoglobulin J region sequences (**I** library). Together the clusters with differentially expressed **A** and **C** sequences had on the average 1.72-fold higher number of I neighbors than the rest (p<1e-7). (**Supplementary Data Sheet 2**)

Correlating degree, public mimotope (**G**), idiotope (**I**), and background sequence (**B**) neighbor counts yielded partial correlations which were significant and positive for degree and **G** (0.862, p<1e-7) and **I** and **G** (0.196, p<1e-7), and negligibly negative between **I** and degree (-0.04, p<1e-7). Thus, for the whole graph the potential idiotope neighbor counts correlated with the public mimotope neighbor counts independent of degree or library bias. Similar results were obtained with regression models (**Supplementary Data Sheet 2**). Controlling for the sequence bias: 1) the mimotopes from both APS enriched (**A**) or depleted (**C**) specificities had a higher number of **I** neighbors than average, 2) the **G** neighbors were less than average for the **A** mimotopes and more than average for the **C** mimotopes, 3) controlling for the dependence of **I** on **G, A** mimotopes had relatively higher number of **I** neighbors than **C** mimotopes (**Supplementary Data Sheet 2****,** **Supplementary Figure 8**).

**Figure 8:**
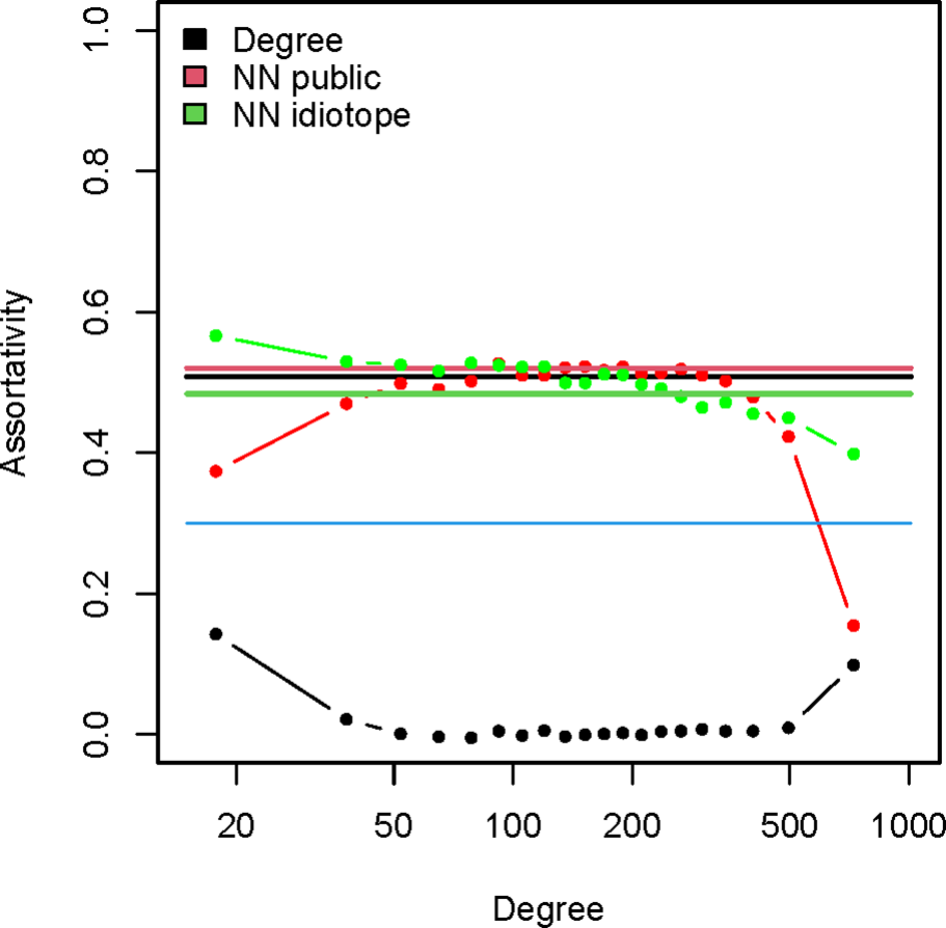
Assortativity of the igome graph. The straight lines indicate the overall assortativity for the graph for the degree (black), number of public mimotope neighbors (red) and number of putative idiotope neighbors (green). The dotted lines represent the respective assortativity values for induced subgraphs stratified by degree. The vertices were sorted in 20 bins by degree and these groups were used to generate induced subgraphs. The mean degree of each group is on the X axis.

The assortativity related to the **I** mapping (0.48) was very high and comparable to the degree assortativity (0.51). Thus, vertices with many idiotope neighbors are connected preferentially to other vertices with many **I** neighbors. The same was true of the assortativity of the **G** mapping which was even higher – 0.52. Since it was possible that this simply reflects the degree assortativity because the numbers of **I** and **G** neighbors depended on the degree, the vertices were split into 20 categories depending on their degree and the assortativity of the subgraphs induced by these vertex sets was calculated (**Figure 8**). While, as expected, this fragmentation of the graph eliminated the degree assortativity it did not affect significantly the **I** and **G** assortativity. Therefore, the observed preferential cross-reactivity between idiotypically connected public specificities was not an artifact of titer of the reactivity.

Thus, the changes in the IgM repertoire in APS were found in compartments with a higher probability for idiotypic connectivity. The specificities lost in APS were more related while those enriched were less related to the public repertoire compared to the unchanged specificities. Although the enriched specificities (**A**) appeared to have slightly less idiotypic neighbors than those lost in APS (**C**), relative to their low public quality they had an unexpectedly high number of idiotypic neighbors (**Supplementary Figure 8**).

**Figure 9:**
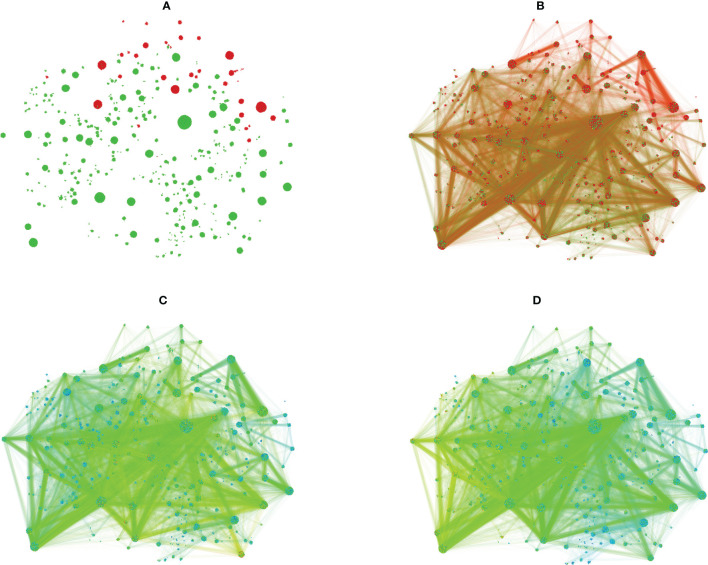
Subgraph including the sequences in the significant clusters produced using the Gephi software. **(A)** – edges are hidden, coloring by type of NGcl or WGcl cluster, red – **A,** green - **C** A vertex gets the color of the cluster it belongs to, e.g. - a C library sequence in a cluster with predominance of A sequences will be red and not green. The spots are actually very dense clusters of vertices (sequences). The clustering shown is a result of application of the OpenOrd layout algorithm (51) and is unsupervised with respect to the clustering of the data in NGcl and WGcl sets of clusters. Therefore, the clusters seen in the plot are not the original NGcl or WGcl clusters, they are split in a different way so, for instance, there are more **A** clusters but the overall grouping is preserved so the clusters shown here remain of a homogenous color. **(B)** – the same layout with edges. Unlike the previous panel, here the coloring of each vertex and the adjacent edges is according to library the respective sequence belongs to, red – **A**, green – **(C)** Above and below are seen regions with high predominance of resp. **A** and **C** vertices. The majority of the **C** clusters have also considerable numbers of **A** vertices so, for instance, clusters which appear green in **(A)** here have both red and green spots but the cluster they belong to has a significant predominance of **C** sequences hence the overall green color in **(A)**. **(C)** – the same layout as in **(B)** color coded for number of **G** (public) sequences in the neighborhood of each vertex. The color ranges from blue (lowest) through yellow to red (highest) number. **(D)** – the same layout and color scheme as in **(C)** but the color code refers to the number of **I** (idiotope) sequences in the neighborhood of each vertex. Interestingly, the most idiotypically relevant clusters are away from the regions with higher diagnostic specificity.

The counts of the public (**G**) and the idiotope (**I**) neighbors of the sequences in **A** and **C** clusters were mapped visually to a subgraph induced by all unique members of the clusters with a significant **A** or **C** overexpression in both WGcl and NGcl (n=17 831) (**Figure 9**). It shows a large overlap between parts with high **G** and **I** representation and also some clusters with a specific predominance of **G** or **I** association. Interestingly, the regions with extreme ratios for **A** or **C** had a lower **I** representation.

#### 3.3.5 Biological Properties of the APS Related Mimotope Sequences

Antibody repertoire differences in autoimmunity versus healthy controls are expected to be related to pathognomic specificities. It seems it is not necessarily so for IgM. As seen in the results reported here the signature changes represent rather a changed balance of specificities related to public clones and idiotypic interactions. Nevertheless, it is important to know if particular autoreactivities are involved in the maintenance of this homeostasis in health and autoimmunity. To gain more insight in the structural basis for the defined clusters of mimotopes (**A** and **C** specific in NGcl and WGcl) statistical sequence models were built in the form of position-specific scoring matrices (PSSM) for each cluster. Logos of the models are shown in **Supplementary Figure 9** and a ROC curve illustrating the capacity of the models in general to differentiate clusters is shown in **Supplementary Figure 10** (area under the curve, AUC=0.995). For a comparison, MEME motifs were extracted for the **A** and **C** groups of clusters after pooling all sequences from the clusters so the algorithm was not guided by our clustering (**Supplementary Data Sheets 3** and **4**).

The models of the **A** clusters (both from NGcl and WGcl) were used to look for linear epitopes in the Immune Epitope Database [http://www.iedb.org/, (45)] which could be related to the defined clusters. Out of 136 884 linear epitopes longer than 6 residues, 369 were identified as related to the APS associated mimotope clusters (**Supplementary Data Sheet 5**) coming from 79 different species including food and human antigens. The sample of antigens seemed unrelated to immune memory since among the most represented were rare infections not encountered in the targeted population as onchocerciasis, trypanosomiasis, MERS, and SARS-CoV1 (the samples are collected before the COVID-19 pandemic).

Although the PSSM models were ideally suited for the search of homologous natural sequences, the search in the human proteome (116 263 protein sequences) yielded more than 25 000 hits. To increase maximally the stringency of the search given the short length of the mimotopes only exact matches in the proteome of any of the mimotopes in each cluster were searched for. This search yielded 1072 mimotope sequences found in 7 519 different proteins (1367 after collapsing the isoforms, **Supplementary Data Sheet 6**). Only one sequence was found in vimentin and none in β2GP1 or annexin 5. These are the major autoantigens for IgG antibodies in APS. Applying the graph analysis, 7-mers from these three proteins were mapped to the previously described (**Figure 9**) graph of significant clusters (**Supplementary Figure 11**). As expected, there were numerous sequences which mapped to the neighborhoods of differentially expressed reactivities but there was no clear distinction between **A** and **C** clusters. Furthermore, similar mappings can be obtained with most proteins in the proteome (data not shown).

Larger clusters had a higher number of peptidome hits per sequence (R^2 =^ 0.16, p=0.00173) and independently also a weak but significant correlation with mean idiotope neighbor counts - **I** (R^2 =^ 0.14, p=0.0074). The peptidome hits among both **A** and **C** calls had a higher mean number of **I** neighbors than the rest of **A** and **C.** The peptidome hits among **A** calls had a lower mean number of **G** neighbors than the rest of **A** while the peptidome hits among **C** calls had a higher mean number of **G** neighbors than the rest of **C** (**Supplementary File 2**). Thus, the detectable autoreactivity of IgM involved in APS expressed stronger the characteristic of the respective **A** or **C** calls – a common high idiotypic connectivity and a contrasting relation to public reactivities (**Supplementary Figure 8**).

Some of the mimotope sequences of **A** and **C** were found identical to parts of some J regions. Among the J regions, 55 had sequences found in **A** and 705 – in **C** mimotopes. These J regions were characterized by slightly longer sequences (mean for the pooled J regions with **A** or **C** sequences – 16.2 residues) than the mean of the assayed J regions (13.9 residues) (p<0.05, Man-Whitney U test). The **C** sequence carrying J regions had an insignificantly higher number of residues than the **A-**associated ones (16.3 and 15.5). The **C**-associated J regions had a significantly higher frequency of the residues D, S, and Y and lower of A, R, N, I, P, T, and V than the **A**-associated J regions (**Supplementary Figure 12**). These parameters characterize the **C** mimotope sequence containing J regions as probably derived from expanded naïve B cells (52).

**Figure 10:**
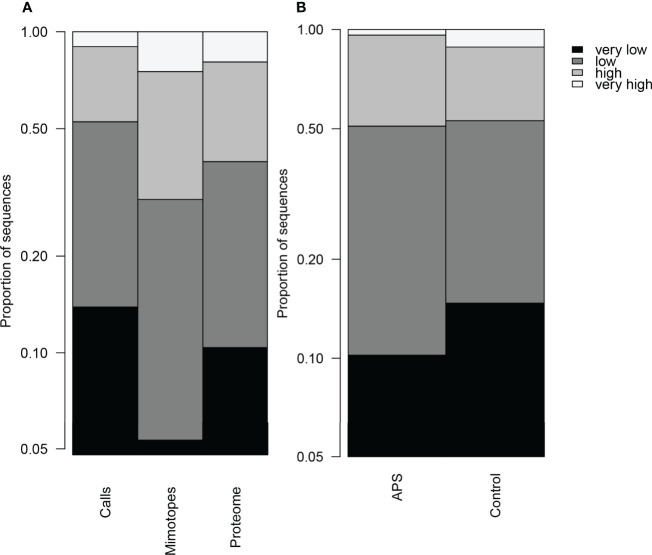
Distribution of peptide sequences by entropy, the discrete entropy levels of 7-mer sequences calculated using the bias-corrected maximum likelihood method were pooled into 4 groups: very low – below 1.57 nats (typically at least 3 pairs or more repetitions), low - between 1.57 and 2 nats (2 pairs or a triplet), high – 2.1 nats (one pair), very high – 2.37 nats (7 different letters). **(A)** The proteome has low entropy skewed distribution relative to the mimotope library. The mimotopes found in the proteome sequences had an even stronger low entropy bias. **(B)** Among the differentially expressed reactivities, those over expressed in APS had lower proportion of lowest entropy sequences than those under expressed.

The numerous mimotope sequences found in self-proteins were often in low complexity regions. The distribution of the sequences by entropy was different between the mimotope library and the human peptidome (Chi-square test, p<1e-7, **Figure 10A**). Low entropy sequences were a higher proportion in the peptidome than in the mimotope library but the mimotopes found in the peptidome had an even higher proportion of low entropy sequences than the peptidome. The distribution by entropy was different also for the **A** and **C** clusters (**Figure 10B**) with the **C** clusters having more extreme distribution and accounting for more of the low complexity calls. These findings indicate that the physiological autoreactivity of IgM may be preferentially directed to low complexity regions.

Altogether, the graph representation of the IgM Igome suggests that about 0.5% of the IgM specificities in healthy donors may be underexpressed in APS. These are public, mostly idiotypically connected clones which are often cross-reactive with low complexity regions on autoantigens. The 7-fold fewer IgM reactivities overexpressed in APS are also idiotypically connected but are less related to the public repertoire and cross-react less with low complexity sequences.

## 4 Discussion

Investigation of the vast diversity of the antibody repertoires is possible by direct sequencing of single cells’ BCR or by indirect methods based on high throughput generation of mimotopes (36, 53). The latter yield big data sets of peptide sequences reflecting the repertoire reactivities referred to as Igome (36, 39). The fact that small linear peptides mimic the mostly conformational B-cell epitopes is a limitation. Yet, a rich peptide library should provide enough sequences to approximate the real B-cell epitopes besides representing linear parts of them (54). The current study applies this approach to compare the IgM repertoire in APS patients versus healthy controls.

For using the Igome to map the repertoire at least an approximation of the individual specificities’ footprints is needed. Each B cell clone is hypothesized to be reflected in several subsets of related mimotopes. It has been known for some time that a single antibody paratope can bind unrelated sequences (55, 56). Furthermore, with an increasing number of amino-acid substitutions a single linear epitope loses affinity incrementally (up to a point) (57). Thus, for each mimotope, a family of closely related sequences exists that bind to the same antibody with a detectable affinity. Still, a paratope should be imprinted in multiple groups like that.

To explore these relations better, data were retrieved from the Biopanning Data Bank (http://i.uestc.edu.cn/bdb/) (41) generated in 49 experiments which had used monoclonals to probe the same PDL for mimotopes. Most isospecific sets of mimotopes consisted of a large group of highly related sequences plus a small number of sequences unrelated to any of the rest. On the other hand, when the deep panning technique was used to create as big libraries of mimotopes of the same monoclonal as possible, no clear limit of the number of selected diverse sequences was observed but they were found to cluster in 14 to 48 motifs (39). Together this evidence points to a hypothetical topology of the footprint of a single antibody in the Igome. It seems that on average a single antibody specificity is represented by about 30 clusters of similar sequences. These clusters may vary in size dependent on the diversity of the starting peptide library, the length of the sequences, the concentration of the selecting antibodies, the specificity needed, the stringency of selection, etc.

Using ROC analysis, we found that for 7-mer mimotopes 4 common residues is the optimal cut-off for distinguishing pairs of isospecific mimotopes with similar sequences from random sequence pairs. The sensitivity, though, was low because only the group of similar sequences in each isospecific set defined the profile. The remaining unrelated sequences could be representatives of the other polyspecificity clusters remaining below the threshold of selection because of lower affinity. With these considerations, a more stringent criterion was applied – LCS of 5 common residues out of 7 yielding a specificity of over 0.9 and effectively concentrating on the cores of the polyspecificity clusters of mimotopes.

This view of the topology of the Igome served as a basis for its formal representation as a graph. The concept is similar to the one used recently to analyze repertoire sequencing data. Like the network of immunoglobulin sequences studied by Miho et al. (20) the APS/healthy Igome graph had an exponential degree distribution. It is characteristic for evolving networks with random attachment (50). A possible mechanism for the attachment of the new clones to the network is selection and recruitment to the repertoire by the same set of antigens. It is interesting that the two images of the repertoire with different yet related mechanisms for evolution (sequence mutations (20) vs footprints of selecting antigens) should have similar graph characteristics.

The hypothesis that the IgM repertoire is an evolving network of progressively but randomly attached (coselected) poly-specificities is in line with several existing notions of the properties of the repertoire (37, 58, 59). It is also a possible explanation for the high density of the IgM Igome graph although the IgM repertoire is the most diverse (60). Another reason for the high density of the graph could be the degeneracy of the probing library due to the short sequence length. On the other hand, probing with the shortest possible peptides allows exploring a greater part of the specificity space because of the workable 10^9^ diversity of the 7-mers with a lower resolution as a trade-off. The actual mimotope sequence space explored, though, proved to be only 10% of the set of all 7-mer peptides due to biases in the used PDL.

The most intriguing finding about the differentially expressed specificities was the prevalence of those with reduced over those with increased expression in APS. Throughout this report, the term specificity is used under the condition that the observed clusters of mimotopes reflect at least partially the footprints of actual clones. The argument for that was given above but it is necessary to underlie the surrogate nature of these antibody images. The low number of clusters observed (in the order of 10^3^-10^4^) indicates that these are most probably coarse groupings of cross-reactivities between multiple IgM clones. This notion is closer to the proposed concept of antigenic field (61) but rather represents its complementary “specificity field”. Thus, the meaning of specificity, as used here, is somewhat decoupled from the individual IgM clones and rather represents a property of the repertoire. As a unite of the Igome, a specificity cluster is also related to the resolution of the method. At the limit, the fine-grained resolution of such a set is defined by the neighborhoods of each vertex in the graph. These mimotope units are overlapping not unlike the clonal specificities but this fact may complicate the formal representation. Instead, to get an image which is easier to interpret at the expense of a lower resolution, the partitions of the graph in clusters were analyzed further. Thus, the observed changes in the IgM repertoire probably affect multiple clones cross-reactive with the observed specificities. The proportion of such classes of cross-reactivities found changed in APS (20 out of 3585 for the whole graph) suggests that the observable changes of the IgM repertoire affect less than 1% of the clones.

The diagnostic role of IgM antibodies in APS has been a subject of debate. The recent findings show that although IgM APLA may be detectable and clinically useful (62) they have an independent diagnostic value in about 12% of the patients only (62, 63). Our findings draw the focus to the repertoire changes in IgM which do not necessarily affect APLA IgM but other clones which are somehow related to them on a systemic level. Indeed, even the specificities found over-represented in APS do not contain known motifs of APLA mimotopes (64, 65). The peptidome hits contain a single full-length peptide found in a known APS auto-antigen - vimentin ^428^LDSLPLV^434^. When β2GPI, annexin 5 and vimentin were mapped to the graph of differentially expressed specificities numerous sequences from each antigen were found in the neighborhoods of the mimotopes. At the same time this potential cross-reactivity was not different quantitatively from that of a random selection of 1200 other human proteins. Besides, the predominant change seems to be rather the loss of specificities in the IgM repertoire.

A key property of the antibody repertoires is the presence of public clones and/or specificities. Public specificities are the more general phenomenon because they can be related to public variable regions or to a somatic evolution convergent on a reactivity to the same epitope. In humans, the probability of CDR-H3:CDR-L3 combination recurrence is found to be 3x10^-5^ (out of 179 000 sequences) (66) while in mice identical CDR-H3 sequences in at least two individuals were found in 23% of the naïve B cells (out of 4x10^6^ VH sequences from 19 mice) (67). Later it was shown that in humans the percentage of IgM public clones depends on the size of the sequence set (68) but the estimates the authors give span the whole range of 10^-4^-10^-1^. Mining a huge dataset, Yang et al. find also truly public IgM germline sequences dependent only on combinatorics and selection (68). The image of antibody public reactivity was observed long ago when physiological autoreactivity was probed yielding consistent repertoire profiles between healthy individuals (69, 70). The gradual reconstitution of this public repertoire after myeloablative treatment for lymphoma was also described (71) Recently, an attempt was made to define the human public Igome using IgM from 10 000 healthy donors (21). The data of two similar experiments were pooled to yield 2 796 484 public IgM mimotope sequences. This allowed mapping the APS-related repertoire to the public IgM Igome.

Interestingly, the most sequence similarities in the human proteome to IgM mimotopes were in J regions of other antibodies (21). Operationally, an epitope found in another antibody’s J region corresponds to the somewhat marginalized concept of an idiotope (72). Thus, if our previous findings (21) hold true, relating pathology-associated Igome changes to public reactivities should naturally be tied also to idiotypic relations involving HCDR3. To investigate this, human immunoglobulin J regions fragments were projected onto the IgM Igome graph. An abundance of similarities between J regions and IgM mimotopes was seen. The number of J neighbors to an Igome sequence ranged from 0 to 21 352. All differentially expressed reactivities in APS were better related to idiotopes than the average for an Igome sequence. On the other hand, the underexpressed reactivities correlated more with public mimotopes than the overexpressed. The sequences of the mimotopes lost in APS were found in longer J regions poor in positive charges, characteristics typical of expanded naïve B cell clones (52). The findings allow hypothesizing an altered idiotypic selection of the emerging repertoire in APS. The evidence of positive selection of the emergent repertoire of B cells (16, 23, 52, 73, 74) with a possible participation of idiotypic interactions has long been discussed (75–77). It would seem a plausible mechanism for the observable idiotypic relations. A loss of complementary anti-idiotypic antibodies has been observed also in other autoimmune diseases (33, 78, 79). On the other hand, the role of IgM in autoimmunity may be more complex as in the so called adaptive tolerance (80) where high affinity memory IgM controls autoimmunity to insulin but our findings seem less related to this mechanism.

The most interesting finding of cross-reactivity of APS associated specificities with the peptidome was the abundance of low complexity targets. Repetitive sequences are often part of disordered proteins and both structural classes are found to contain often linear B cell epitopes of antibody responses to infectious agents (81, 82) but also a source of possible autoimmunity causing cross-reactivity (83, 84) and even used as decoys to thwart the antibody response (85). It would be interesting to explore further the role of a low complexity self-epitopes in APS.

In summary, an Igome image of the IgM repertoire formalized as a graph detected effectively global deviations in the APS-associated repertoires. These changes bear the sign of system level effects possibly due to a pressure from the overexpressed pathological antibodies on the selection of the emergent repertoire. This novel insight could be a potential source of future theranostic targets.

## Data Availability Statement

The datasets presented in this study can be found in online repositories. The names of the repository/repositories and accession number(s) can be found below: https://github.com/ansts/aPLAb1.

## Ethics Statement

The studies involving human participants were reviewed and approved by Human Studies Ethical Commission at The Institute of Biology and Immunology of reproduction, Bulgarian Academy of Sciences. The patients/participants provided their written informed consent to participate in this study.

## Author Contributions

SP participated in conceptualizing the study, organized and managed the sample collection, organized and ran the phage display experiments, managed the sequencing tasks and the data collection and storage, and processed the raw data. LB ran the sequencing of the phage libraries. GE and AS collected and organized the patient samples. ES ran the immunochemistry and phage display. VS participated in conceptualizing the study, the analysis of the results, and the preparation of the manuscript. PP participated in conceptualizing the graph representation and the analysis of the results. AP conceptualized the project, analyzed the results including all the in-silico analysis, supervised experiments except for the sequencing as well as the overall project execution, and participated in the preparation of the manuscript. All authors contributed to the article and approved the submitted version.

## Conflict of Interest

Author VS is employed by ICON plc, Sofia.

The remaining authors declare that the research was conducted in the absence of any commercial or financial relationships that could be construed as a potential conflict of interest.

## Publisher’s Note

All claims expressed in this article are solely those of the authors and do not necessarily represent those of their affiliated organizations, or those of the publisher, the editors and the reviewers. Any product that may be evaluated in this article, or claim that may be made by its manufacturer, is not guaranteed or endorsed by the publisher.

## Funding

This work was performed with the support of the Bulgarian Fund for Scientific Research Grants KP-06-N21-14/2018, KP-06-N37-24/2019 and DN 01-11/2016.

## Supplementary Material

Supplementary Figure 1 A “distance” matrix illustrated by longest common subsequences between the mimotopes of a single monoclonal anti-Fas antibody SA-7 (based on {Abbasova, 2007 #24698}).

Supplementary Figure 2 Distribution of the sizes (equal also to the degree of the central vertex) of all neighborhoods (gray) and of the differentially represented neighborhoods (red).

Supplementary Figure 3 Distribution of the sizes (equal also to the degree of the central vertex) of neighborhoods overrepresenting control library (gray) and APS library (red).

Supplementary Figure 4 Analysis of the distribution of G library mimotope neighbors in the significant neighborhoods. X – log odds ratio of A to C mimotopes in the neighborhood. Above – histogram of the significant neighborhoods by LOR. Bellow – distribution and plots of the LOR of global mimotopes to neighborhood sizes relative to the groups of global mimotopes formed on the basis of their overlap with C and A mimotopes. Global – global mimotopes not found in A or C; Global & APLS, Global & Cntr and Global and All – sequences common to G, A and C libraries in the different combinations thereof.

Supplementary Figure 5 Volcano plot illustrating the differential representation of control or APS specific mimotope sequences in each cluster of sequences from the whole graph (red – A or green - C).

Supplementary Figure 6 Comparison of mean degree (left) and mean eigen centrality (right) within the whole igome graph of the 4 groups of clusters (AllAPLS – A clusters in the whole graph clustering, AllCntr – C clusters in the whole graph clustering, NAPLS - A clusters in the significant neighborhoods subgraph, NCntr – C clusters in the significant neighborhoods subgraph). The means are compared to means bootstrapped over 1000 random samples of the observed clusters (red lines indicating the 5th and 95th percentile of the distribution). In the whole graph A clusters have clearly higher mean degree and eigen centrality than C clusters. This effect is lost or even reversed (for eigen centrality) in the significant neighborhoods-based clusters probably because the respective subgraph focused on the denser parts of the whole graph so the C clusters have higher mean degree in the neighborhood clusters than in the whole graph.

Supplementary Figure 7 Comparison of the modularity values of the induced subgraphs based on each of the four groups of clusters (A and C in NGcl and A and C in WGcl – grey bars) with the distribution of modularity in similar groups of clusters bootstrapped over 1000 samples from the available WGcl clusters (red boxplots). The quantile of the observed modularity (the bar) relative to the bootstrapped distribution (boxplot) is inserted above each bar.

Supplementary Figure 8 Specificities enriched or lost in APS have different global public G and idiotope Id characteristics. The plot illustrates the dependence of number of idiotope neighbors on the number of global public neighbors for the stratified mimotopes set. The values of Id and G represent the residuals (effectively – z scores of the residuals) of the regression respectively of Id and G on background sequence distribution B. In other words, the effect of the sequence bias of the phage display library on the density of the graph is removed from the represented regression and distributions. Kernel density was estimated to produce the 10th and 90th percentile contour of the distributions. The points indicate the respective means with the whiskers indicating 10 SEM (multiplied to make them visible and for better significance). The blue line indicates the overall linear regression of the non-stratified set of values. Note that although the specificities lost in APS have a higher mean number of idiotope neighbors than those enriched, controlling for the Id=f(G) dependence their value is actually lower (closer to the line). This means the IgM specificities enriched in APS have an idiotypic property disproportionately high for their global public property. All calculations are based on the decimal logarithms of the numbers of neighbors of Id, G or B type used instead of the raw counts to stabilize their variance.

Supplementary Figure 9 Logo representations of the statistical models of the A and C clusters. The names are formed from the number of the cluster, the library mimotopes from which predominate and the letter v for the clusters coming from the neighborhood-based subgraph (NGcl).

Supplementary Figure 10 ROC curve illustrating the quality of classification of sequences as belonging to the clusters based on the models of the clusters. Optimal separation occurs at score 8.3 which provides specificity of 0.97 and sensitivity of 0.98 with an area under the curve of 0.995.

Supplementary Figure 11 Mapping the 7-mer sequences from beta2GPI **(A)**, annexin 5 **(B)**, and vimentin **(C)** to the subgraph (shown on **Figure 9A**) of the sequences in the significant clusters. The autoantigen peptides are not directly represented but the number of autoantigen peptides in each neighborhood is mapped as an attribute of the corresponding central vertex. The edges color code reflects the number of autoantigen neighbors of the vertices they connect. Only edges between vertices which have at least one neighbor from the autoantigen are show (deep blue). Edges between vertices with a higher number of neighbors are colored respectively from green to yellow and red. The color of the clusters is the same as in **Figure 9A**) representing the differential expression of the original cluster. Both A and C clusters have numerous neighbors from all three antigens. Interestingly, vimentin which is the only intracellular antigens and has the highest number of cross-reactivities maps to the zone with a higher concentration of idiotypic connections.

Supplementary Figure 12 Comparison between the frequencies of the amino acid residues of the J regions containing control (black and grey) or APLA (red and white) mimotope sequences pooled from WGCl and NGcl clusters. The significant differences are in black and red (Chi square test, p<0.05).
