# Supplementary figures and images for: Restriction of the Global IgM Repertoire in Antiphospholipid Syndrome
Source: Front Immunol. 2022 Apr 13;13:865232. doi: 10.3389/fimmu.2022.865232 (PMC9043687; doi:10.3389/fimmu.2022.865232)

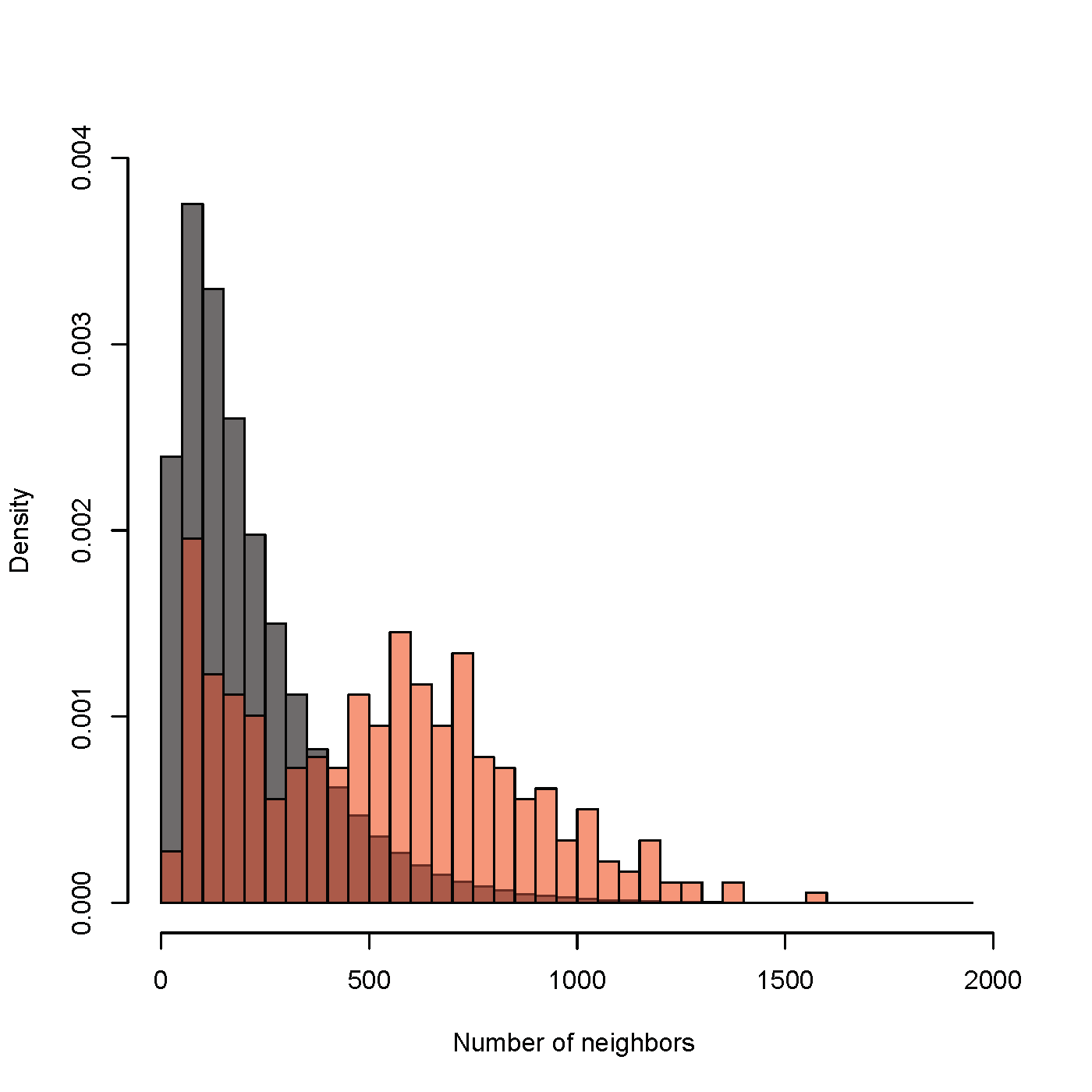

Supplement: Supplementary Figure 2 — Distribution of the sizes (equal also to the degree of the central vertex) of all neighborhoods (gray) and of the differentially represented neighborhoods (red). [file Image_2.png]

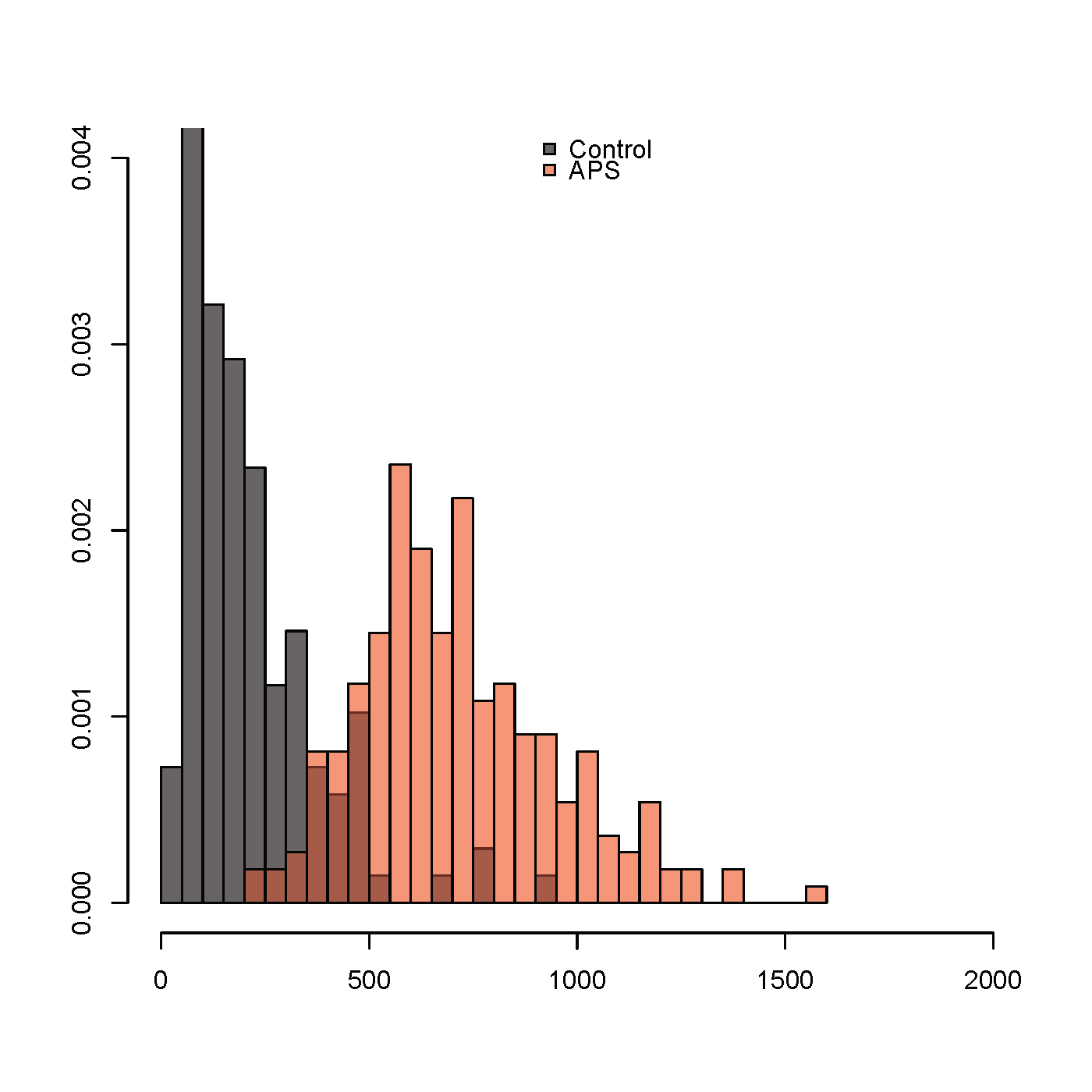

Supplement: Supplementary Figure 3 — Distribution of the sizes (equal also to the degree of the central vertex) of neighborhoods overrepresenting control library (gray) and APS library (red). [file Image_3.png]

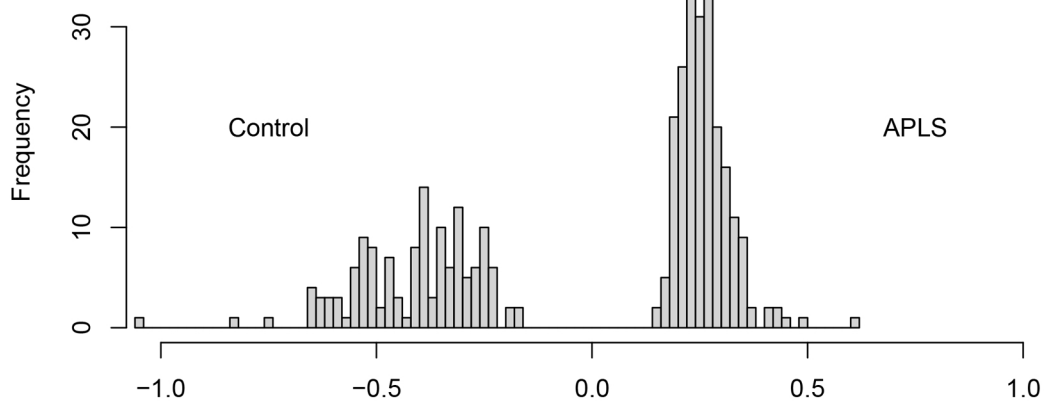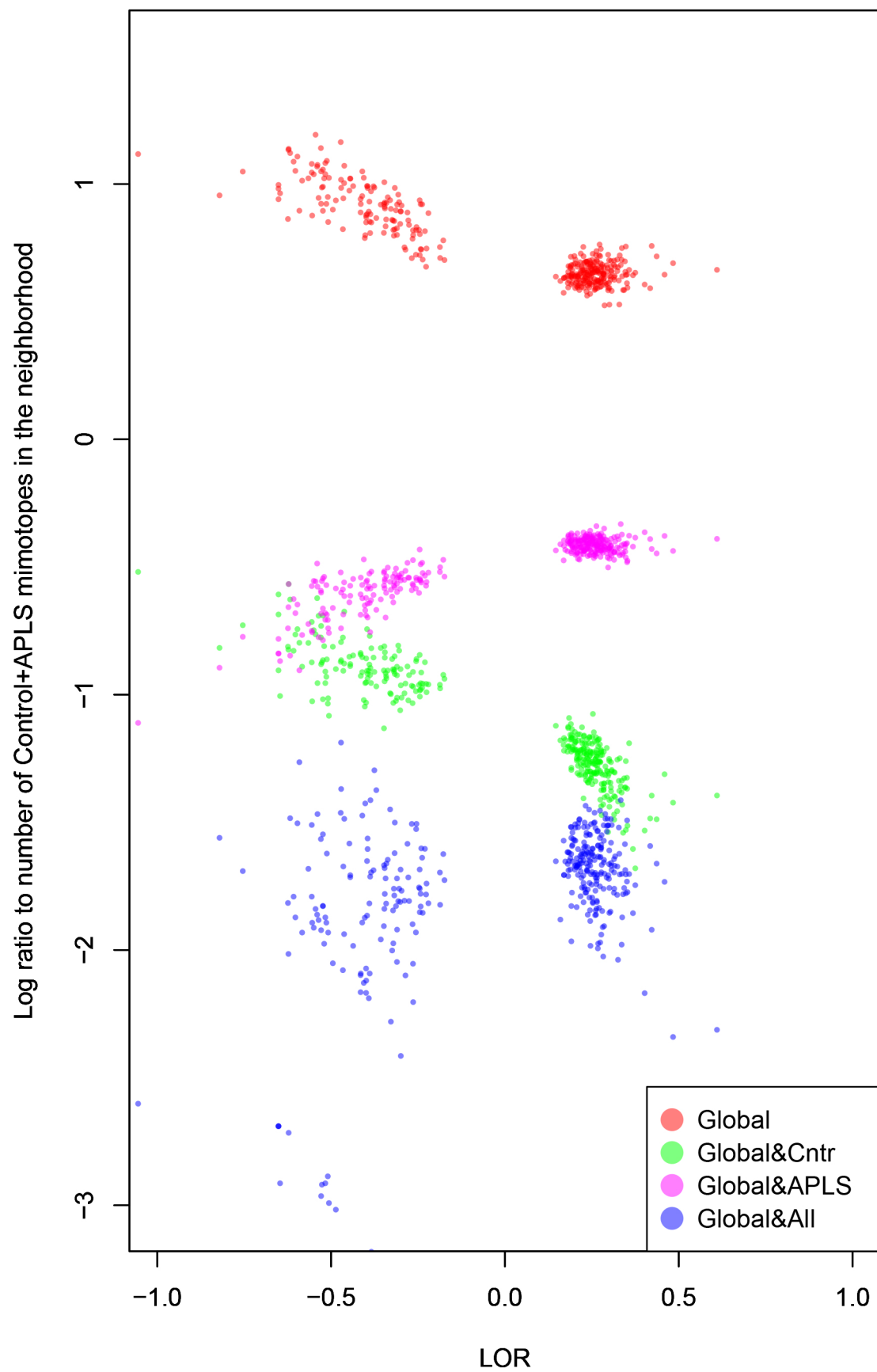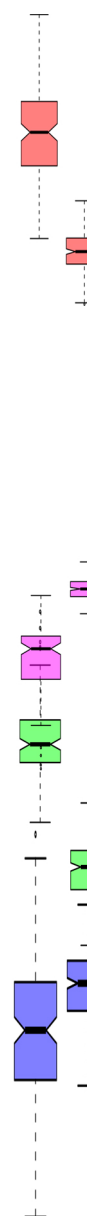

Supplement: Supplementary Figure 4 — Analysis of the distribution of G library mimotope neighbors in the significant neighborhoods. X – log odds ratio of A to C mimotopes in the neighborhood. Above – histogram of the significant neighborhoods by LOR. Bellow – distribution and plots of the LOR of global mimotopes to neighborhood sizes relative to the groups of global mimotopes formed on the basis of their overlap with C and A mimotopes. Global – global mimotopes not found in A or C; Global & APLS, Global & Cntr and Global and All – sequences common to G, A and C libraries in the different combinations thereof. [file Image_4.pdf]

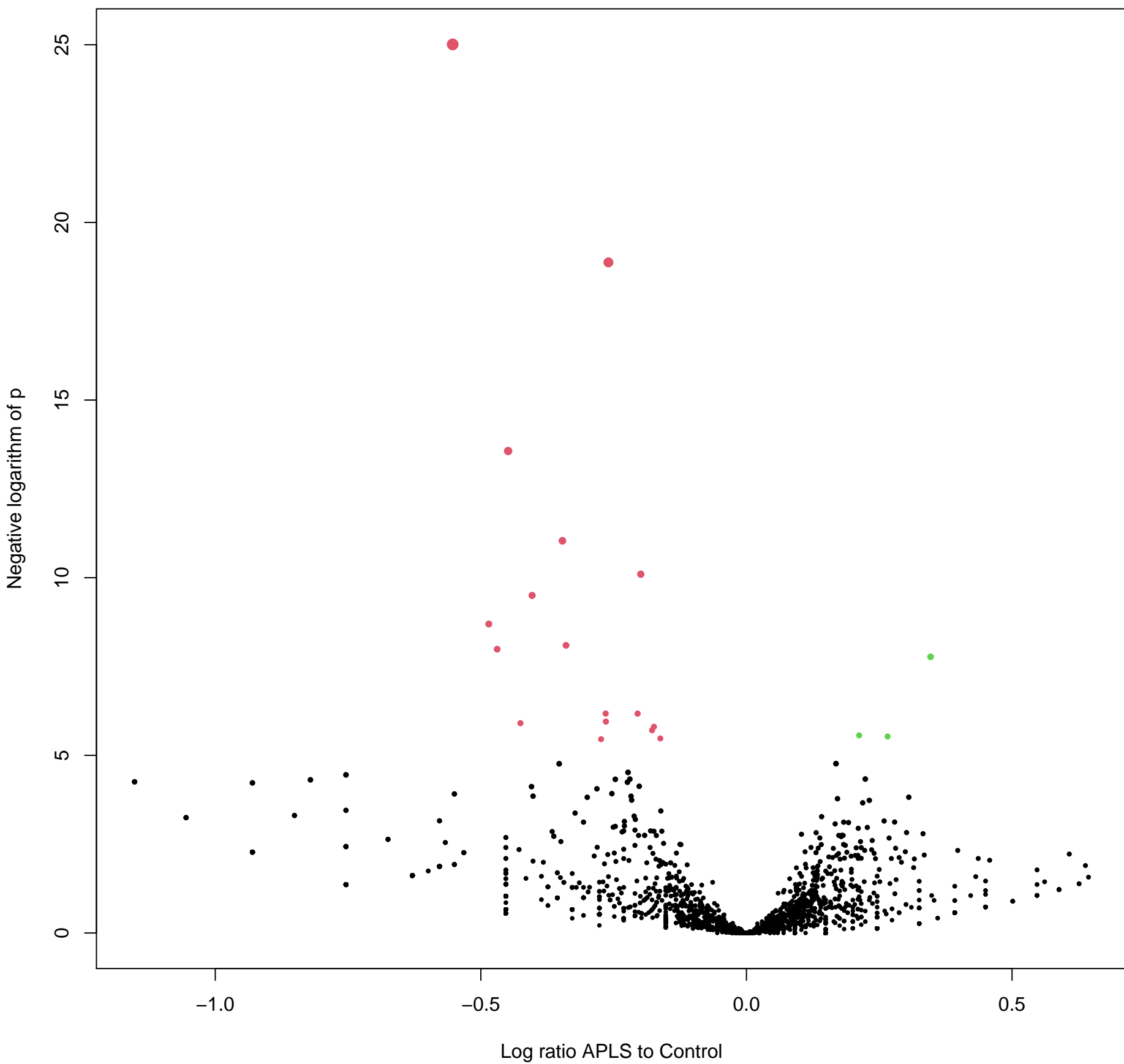

Supplement: Supplementary Figure 5 — Volcano plot illustrating the differential representation of control or APS specific mimotope sequences in each cluster of sequences from the whole graph (red – A or green - C). [file Image_5.pdf]

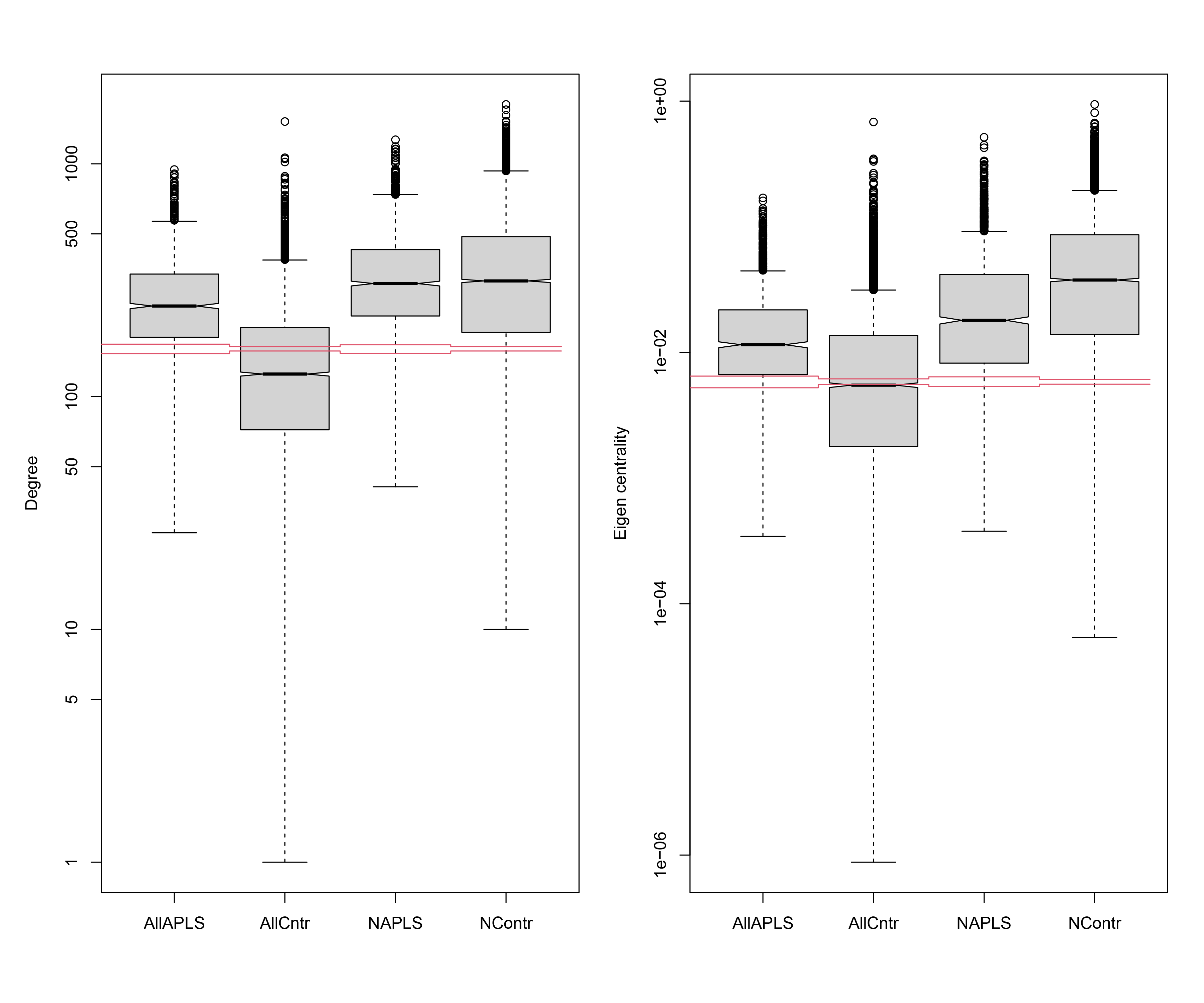

Supplement: Supplementary Figure 6 — Comparison of mean degree (left) and mean eigen centrality (right) within the whole igome graph of the 4 groups of clusters (AllAPLS – A clusters in the whole graph clustering, AllCntr – C clusters in the whole graph clustering, NAPLS - A clusters in the significant neighborhoods subgraph, NCntr – C clusters in the significant neighborhoods subgraph). The means are compared to means bootstrapped over 1000 random samples of the observed clusters (red lines indicating the 5th and 95th percentile of the distribution). In the whole graph A clusters have clearly higher mean degree and eigen centrality than C clusters. This effect is lost or even reversed (for eigen centrality) in the significant neighborhoods-based clusters probably because the respective subgraph focused on the denser parts of the whole graph so the C clusters have higher mean degree in the neighborhood clusters than in the whole graph. [file Image_6.png]

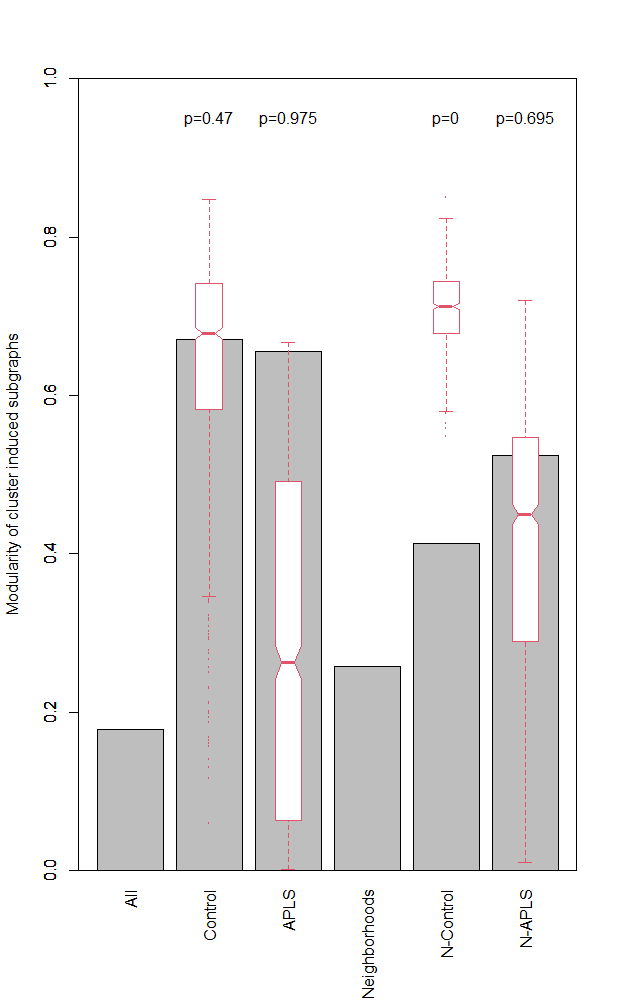

Supplement: Supplementary Figure 7 — Comparison of the modularity values of the induced subgraphs based on each of the four groups of clusters (A and C in NGcl and A and C in WGcl – grey bars) with the distribution of modularity in similar groups of clusters bootstrapped over 1000 samples from the available WGcl clusters (red boxplots). The quantile of the observed modularity (the bar) relative to the bootstrapped distribution (boxplot) is inserted above each bar. [file Image_7.png]

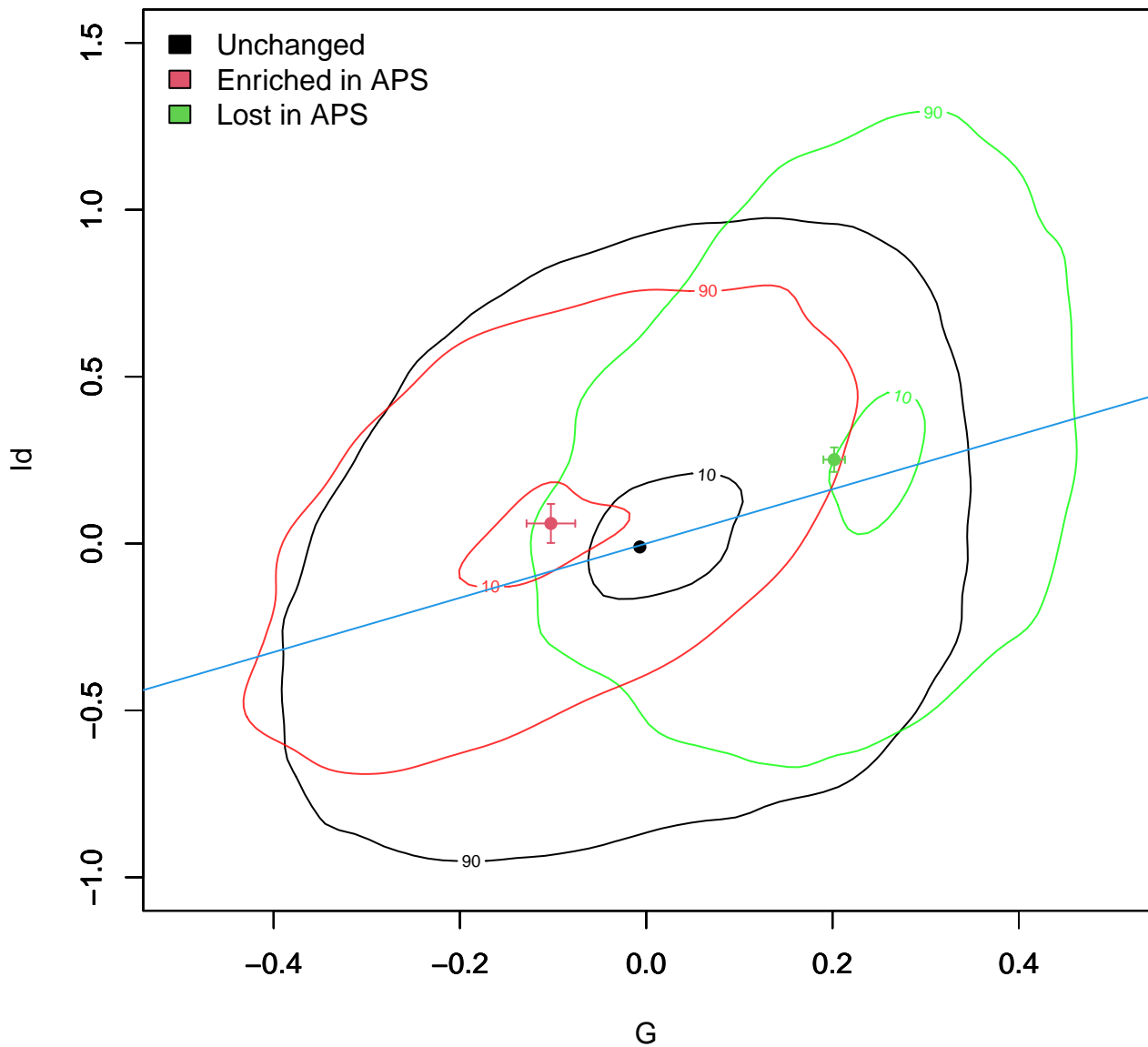

Supplement: Supplementary Figure 8 — Specificities enriched or lost in APS have different global public G and idiotope Id characteristics. The plot illustrates the dependence of number of idiotope neighbors on the number of global public neighbors for the stratified mimotopes set. The values of Id and G represent the residuals (effectively – z scores of the residuals) of the regression respectively of Id and G on background sequence distribution B. In other words, the effect of the sequence bias of the phage display library on the density of the graph is removed from the represented regression and distributions. Kernel density was estimated to produce the 10th and 90th percentile contour of the distributions. The points indicate the respective means with the whiskers indicating 10 SEM (multiplied to make them visible and for better significance). The blue line indicates the overall linear regression of the non-stratified set of values. Note that although the specificities lost in APS have a higher mean number of idiotope neighbors than those enriched, controlling for the Id=f(G) dependence their value is actually lower (closer to the line). This means the IgM specificities enriched in APS have an idiotypic property disproportionately high for their global public property. All calculations are based on the decimal logarithms of the numbers of neighbors of Id, G or B type used instead of the raw counts to stabilize their variance. [file Image_8.pdf]

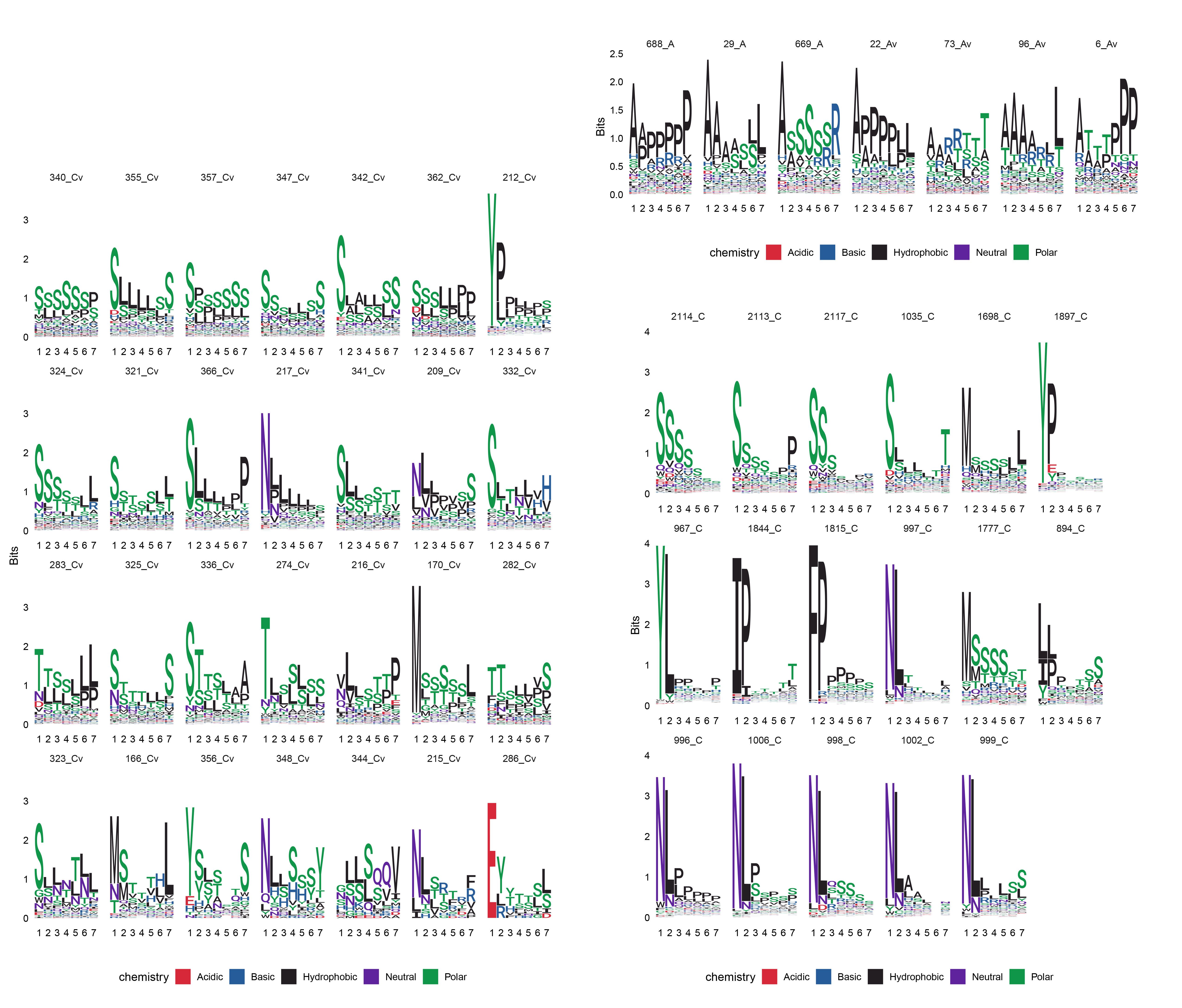

Supplement: Supplementary Figure 9 — Logo representations of the statistical models of the A and C clusters. The names are formed from the number of the cluster, the library mimotopes from which predominate and the letter v for the clusters coming from the neighborhood-based subgraph (NGcl). [file Image_9.png]

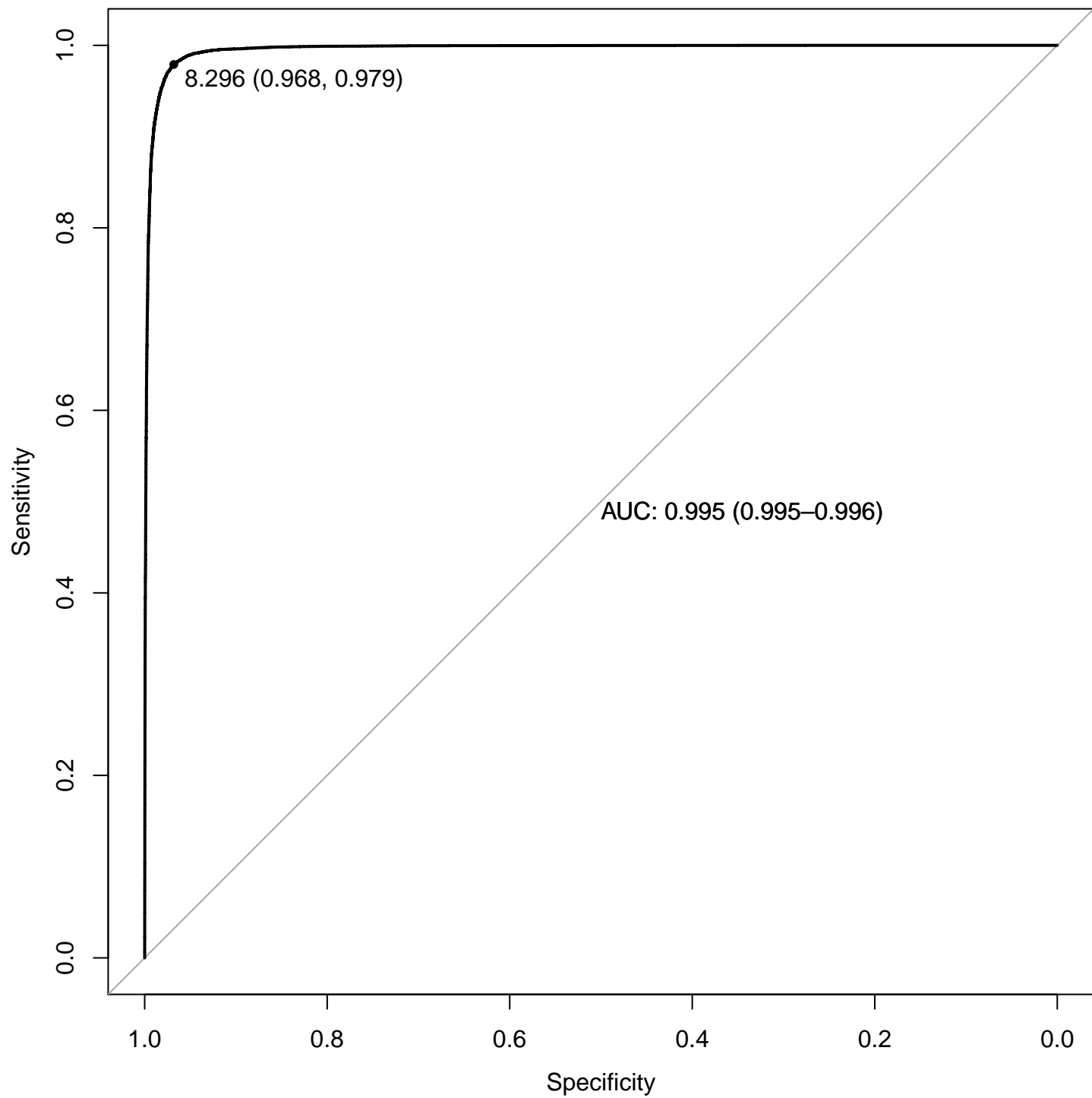

Supplement: Supplementary Figure 10 — ROC curve illustrating the quality of classification of sequences as belonging to the clusters based on the models of the clusters. Optimal separation occurs at score 8.3 which provides specificity of 0.97 and sensitivity of 0.98 with an area under the curve of 0.995. [file Image_10.pdf]

A

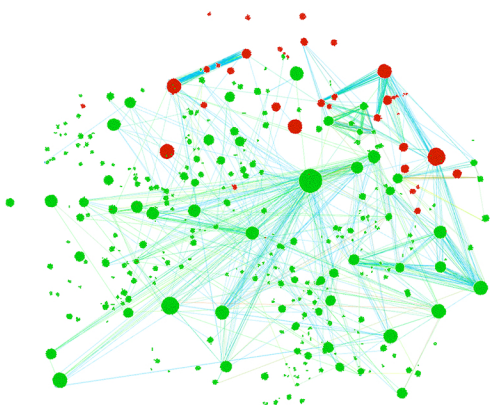

B

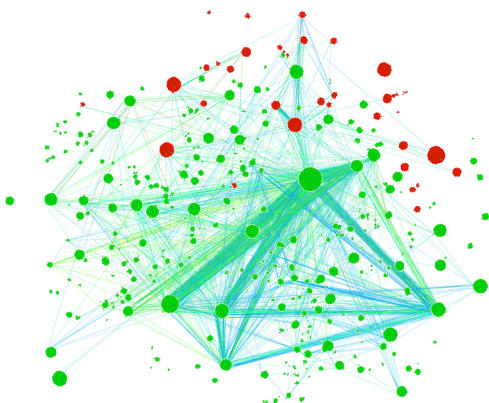

C

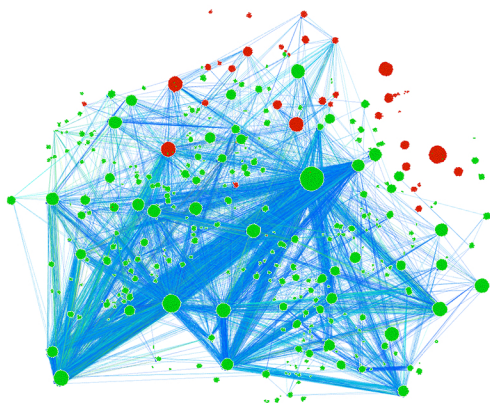

Supplement: Supplementary Figure 11 — Mapping the 7-mer sequences from beta2GPI (A), annexin 5 (B), and vimentin (C) to the subgraph (shown on Figure 9A) of the sequences in the significant clusters. The autoantigen peptides are not directly represented but the number of autoantigen peptides in each neighborhood is mapped as an attribute of the corresponding central vertex. The edges color code reflects the number of autoantigen neighbors of the vertices they connect. Only edges between vertices which have at least one neighbor from the autoantigen are show (deep blue). Edges between vertices with a higher number of neighbors are colored respectively from green to yellow and red. The color of the clusters is the same as in Figure 9A) representing the differential expression of the original cluster. Both A and C clusters have numerous neighbors from all three antigens. Interestingly, vimentin which is the only intracellular antigens and has the highest number of cross-reactivities maps to the zone with a higher concentration of idiotypic connections. [file Image_11.pdf]

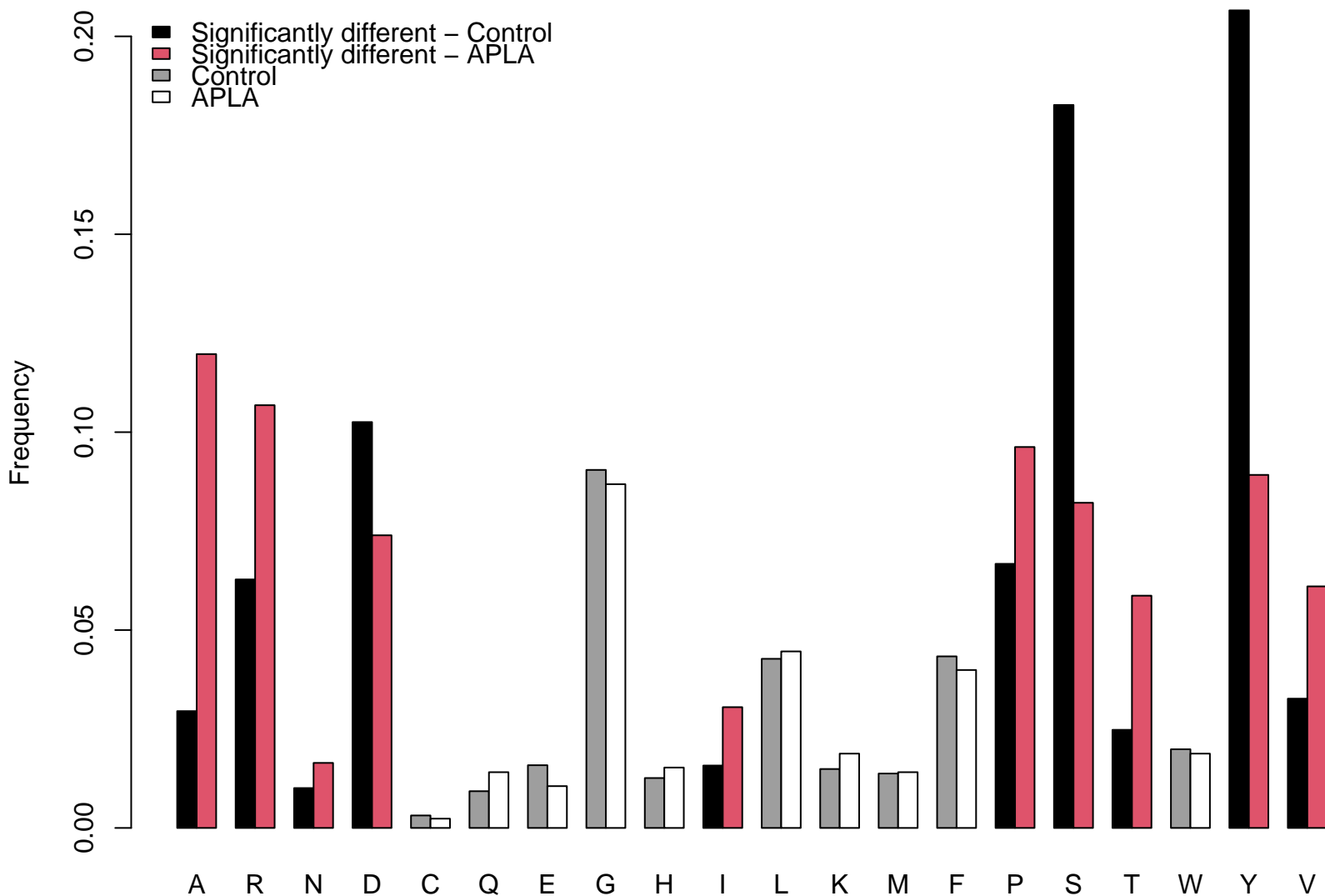

Supplement: Supplementary Figure 12 — Comparison between the frequencies of the amino acid residues of the J regions containing control (black and grey) or APLA (red and white) mimotope sequences pooled from WGCl and NGcl clusters. The significant differences are in black and red (Chi square test, p<0.05). [file Image_12.pdf]
