# Supplementary material for: Restriction of the Global IgM Repertoire in Antiphospholipid Syndrome
Source: Front Immunol. 2022 Apr 13;13:865232. doi: 10.3389/fimmu.2022.865232 (PMC9043687; doi:10.3389/fimmu.2022.865232)
Supplement: Supplementary file 14 [file DataSheet_2.pdf]

Partial correlations

```
$estimate
            D           G          Id          Bg
D   1.00000000  0.86232275 -0.04110607  0.52689141
G   0.86232275  1.00000000  0.19649153 -0.09186836
Id -0.04110607  0.19649153  1.00000000 -0.08540839
Bg  0.52689141 -0.09186836 -0.08540839  1.00000000

$p.value
              D G            Id Bg
D   0.000000e+00 0 5.777371e-146  0
G   0.000000e+00 0  0.000000e+00  0
Id 5.777371e-146 0  0.000000e+00  0
Bg  0.000000e+00 0  0.000000e+00  0

$statistic
           D          G        Id        Bg
D     0.00000 1065.41118 -25.73826 387.83107
G  1065.41118    0.00000 125.37177 -57.71815
Id  -25.73826  125.37177   0.00000 -53.62858
Bg  387.83107  -57.71815 -53.62858   0.00000

$n
[1] 391396

$gp
[1] 2

$method
[1] "pearson"
```

```
##########################################################################################
Call:
lm(formula = D ~ Bg, data = bigtbl)

Residuals:
     Min       1Q   Median       3Q      Max
-1.17786 -0.11721 -0.00671  0.11875  0.81957

Coefficients:
            Estimate Std. Error t value Pr(>|t|)
(Intercept) 0.6192660  0.0012635   490.1   <2e-16 ***
Bg          0.6645671  0.0005271  1260.8   <2e-16 ***
---
Signif. codes:  0 ‘***’ 0.001 ‘**’ 0.01 ‘*’ 0.05 ‘.’ 0.1 ‘ ’ 1

Residual standard error: 0.1732 on 391394 degrees of freedom
Multiple R-squared:  0.8024,  Adjusted R-squared:  0.8024
F-statistic: 1.59e+06 on 1 and 391394 DF,  p-value: < 2.2e-16
```

```
Call:
lm(formula = G ~ Bg, data = bigtbl)

Residuals:
     Min       1Q   Median       3Q      Max 
-1.32295 -0.11941  0.00713  0.12866  0.88567 

Coefficients:
            Estimate Std. Error t value Pr(>|t|)    
(Intercept) 1.6675664  0.0013127    1270   <2e-16 ***
Bg          0.5598197  0.0005476    1022   <2e-16 ***
---
Signif. codes:  0 ‘***’ 0.001 ‘**’ 0.01 ‘*’ 0.05 ‘.’ 0.1 ‘ ’ 1

Residual standard error: 0.1799 on 391394 degrees of freedom
Multiple R-squared:  0.7275,  Adjusted R-squared:  0.7275 
F-statistic: 1.045e+06 on 1 and 391394 DF,  p-value: < 2.2e-16


Call:
lm(formula = Id ~ Bg, data = bigtbl)

Residuals:
     Min       1Q   Median       3Q      Max 
-2.95010 -0.29074  0.01018  0.30470  1.93742 

Coefficients:
           Estimate Std. Error t value Pr(>|t|)    
(Intercept) 2.071126   0.003354   617.5   <2e-16 ***
Bg          0.253243   0.001399   181.0   <2e-16 ***
---
Signif. codes:  0 ‘***’ 0.001 ‘**’ 0.01 ‘*’ 0.05 ‘.’ 0.1 ‘ ’ 1

Residual standard error: 0.4597 on 391394 degrees of freedom
Multiple R-squared:  0.07723,       Adjusted R-squared:  0.07723 
F-statistic: 3.276e+04 on 1 and 391394 DF,  p-value: < 2.2e-16

     Min.   1st Qu.    Median      Mean   3rd Qu.      Max. 
-1.102704 -0.056010 -0.001996  0.000000  0.054495  0.862403 
```

###
Controlling for Bg (in smalltbl Id and G are residuals from their regression on Bg)

```
Call:
glm(formula = Dia ~ (Id + G)^2, family = "binomial", data = smalltbl, 
    subset = smalltbl$Dia != "A")

Deviance Residuals: 
    Min       1Q   Median       3Q      Max  
-2.9741  -0.2740  -0.1690  -0.1038   4.1524  
```

```
Coefficients:
            Estimate Std. Error  z value Pr(>|z|)
(Intercept) -4.17492    0.01500 -278.357  < 2e-16 ***
Id           0.11394    0.03196    3.565 0.000364 ***
G            7.77121    0.06810  114.111  < 2e-16 ***
Id:G         2.86571    0.13211   21.692  < 2e-16 ***
---
Signif. codes:  0 ‘***’ 0.001 ‘**’ 0.01 ‘*’ 0.05 ‘.’ 0.1 ‘ ’ 1

(Dispersion parameter for binomial family taken to be 1)

    Null deviance: 123288  on 387990  degrees of freedom
Residual deviance:  99957  on 387987  degrees of freedom
AIC: 99965

Number of Fisher Scoring iterations: 8


Call:
glm(formula = Dia ~ (Id + G)^2, family = "binomial", data = smalltbl,
    subset = smalltbl$Dia != "C")

Deviance Residuals:
    Min       1Q   Median       3Q      Max
-1.8573  -0.1489  -0.1212  -0.0994   3.5429

Coefficients:
            Estimate Std. Error z value Pr(>|z|)
(Intercept) -4.89362    0.02099 -233.10  < 2e-16 ***
Id           0.73189    0.04563   16.04  < 2e-16 ***
G           -3.55537    0.09839  -36.13  < 2e-16 ***
Id:G        -0.53353    0.18025   -2.96  0.00308 **
---
Signif. codes:  0 ‘***’ 0.001 ‘**’ 0.01 ‘*’ 0.05 ‘.’ 0.1 ‘ ’ 1

(Dispersion parameter for binomial family taken to be 1)

    Null deviance: 38833  on 376969  degrees of freedom
Residual deviance: 37477  on 376966  degrees of freedom
AIC: 37485

Number of Fisher Scoring iterations: 8


Call:
glm(formula = Dia ~ (Id + G)^2, family = "binomial", data = smalltbl,
    subset = smalltbl$Dia != "N")

Deviance Residuals:
    Min       1Q   Median       3Q      Max
-3.6361  -0.3809  -0.1958  -0.0824   3.2246

Coefficients:
             Estimate Std. Error z value Pr(>|z|)
```

```
(Intercept)  -0.73751    0.02890  -25.52   <2e-16 ***
Id            0.99422    0.07177   13.85   <2e-16 ***
G           -12.41913    0.20826  -59.63   <2e-16 ***
Id:G         -4.41346    0.43055  -10.25   <2e-16 ***
---
Signif. codes:  0 ‘***’ 0.001 ‘**’ 0.01 ‘*’ 0.05 ‘.’ 0.1 ‘ ’ 1

(Dispersion parameter for binomial family taken to be 1)

    Null deviance: 17389.2  on 17830  degrees of freedom
Residual deviance:  9297.2  on 17827  degrees of freedom
AIC: 9305.2

Number of Fisher Scoring iterations: 6

##################################################################################
Calls - any differentially expressed

Call:
glm(formula = Calls ~ (Id + G)^2, family = "binomial", data = smalltbl)

Deviance Residuals:
    Min       1Q   Median       3Q      Max
-2.3407  -0.3218  -0.2352  -0.1763   3.6626

Coefficients:
            Estimate Std. Error z value Pr(>|z|)
(Intercept) -3.50154    0.01049 -333.73   <2e-16 ***
Id           0.24587    0.02169   11.34   <2e-16 ***
G            4.82707    0.05352   90.19   <2e-16 ***
Id:G         2.90866    0.09260   31.41   <2e-16 ***
---
Signif. codes:  0 ‘***’ 0.001 ‘**’ 0.01 ‘*’ 0.05 ‘.’ 0.1 ‘ ’ 1

(Dispersion parameter for binomial family taken to be 1)

    Null deviance: 144989  on 391395  degrees of freedom
Residual deviance: 129746  on 391392  degrees of freedom
AIC: 129754

Number of Fisher Scoring iterations: 7

##################################################################################

Peptidome identity hits within A and C dependence on Id and G

Call:
glm(formula = P ~ (Id + G)^2, family = "binomial", data = smalltbl,
    subset = Dia == "A")

Deviance Residuals:
    Min       1Q   Median       3Q      Max
-0.6307  -0.3545  -0.3131  -0.2768   2.7030
```

```
Coefficients:
            Estimate Std. Error z value Pr(>|z|)
(Intercept)  -3.1776     0.1281 -24.796  < 2e-16 ***
Id            1.1766     0.3009   3.910 9.22e-05 ***
G            -1.5596     0.6014  -2.593  0.00951 **
Id:G         -0.0859     1.3813  -0.062  0.95041
---
Signif. codes:  0 ‘***’ 0.001 ‘**’ 0.01 ‘*’ 0.05 ‘.’ 0.1 ‘ ’ 1

(Dispersion parameter for binomial family taken to be 1)

    Null deviance: 1403.0  on 3404  degrees of freedom
Residual deviance: 1380.9  on 3401  degrees of freedom
AIC: 1388.9

Number of Fisher Scoring iterations: 6


Call:
glm(formula = P ~ (Id + G)^2, family = "binomial", data = smalltbl,
    subset = Dia == "C")

Deviance Residuals:
    Min       1Q   Median       3Q      Max
-0.7046  -0.3974  -0.3422  -0.2794   3.3932

Coefficients:
            Estimate Std. Error z value Pr(>|z|)
(Intercept) -3.35543    0.08954 -37.475  < 2e-16 ***
Id           1.33693    0.17063   7.835 4.68e-15 ***
G            1.60055    0.36613   4.372 1.23e-05 ***
Id:G        -1.85178    0.64756  -2.860  0.00424 **
---
Signif. codes:  0 ‘***’ 0.001 ‘**’ 0.01 ‘*’ 0.05 ‘.’ 0.1 ‘ ’ 1

(Dispersion parameter for binomial family taken to be 1)

    Null deviance: 6698.6  on 14425  degrees of freedom
Residual deviance: 6520.9  on 14422  degrees of freedom
AIC: 6528.9

Number of Fisher Scoring iterations: 6
```
