# Supplementary material for: Restriction of the Global IgM Repertoire in Antiphospholipid Syndrome
Source: Front Immunol. 2022 Apr 13;13:865232. doi: 10.3389/fimmu.2022.865232 (PMC9043687; doi:10.3389/fimmu.2022.865232)
Supplement: Supplementary file 15 [file DataSheet_3.pdf]

XSTREME
Motif Discovery and Enrichment Analysis

For further information on how to interpret these results please access https://meme-suite.org/meme/doc/xstreme-output-format.html.
To get a copy of the MEME software please access https://meme-suite.org.

If you use XSTREME in your research, please cite the following paper:
Charles E. Grant and Timothy L. Bailey, "XSTREME: comprehensive motif analysis of biological sequence datasets", *BioRxiv*, 2021.

Motifs | Programs | Input Files | Program information | Summary in TSV Format | Non-redundant Motifs in MEME Text Format

Description

Motifs of mimotopes over-expressed in APS.

Motifs

Enriched motifs (E-value ≤ 0.5 and 3 best STREME motifs).

Expand All Clusters   Collapse All Clusters

Motif Logo	Motif Source	Rank	E-value	Positional Distribution	Matches per Sequence	Similar Known Motifs	Sites
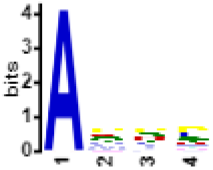	MEME-1 (MEME)	undefined	1.40e-302				Motif Sites in GFF3

Show 1 More ↧

Motif Logo	Motif Source	Rank	E-value	Positional Distribution	Matches per Sequence	Similar Known Motifs	Sites
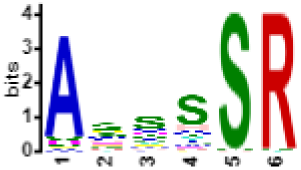	2-ASSSSR (STREME)	undefined	1.67e-013				Motif Sites in GFF3

Motif Logo	Motif Source	Rank	E-value	Positional Distribution	Matches per Sequence	Similar Known Motifs	Sites
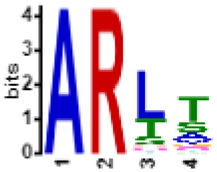	3-ARLT (STREME)	undefined	7.66e-013				Motif Sites in GFF3

Motif Logo	Motif Source	Rank	E-value	Positional Distribution	Matches per Sequence	Similar Known Motifs	Sites
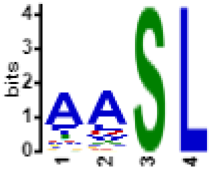	4-AASL (STREME)	undefined	2.31e-007			STATHMIN_1 (PS00563)	Motif Sites in GFF3

Motif Logo	Motif Source	Rank	E-value	Positional Distribution	Matches per Sequence	Similar Known Motifs	Sites
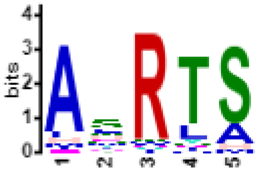	5-ASRTS (STREME)	undefined	4.89e-005				Motif Sites in GFF3

Show 1 More ↧

Motif Logo	Motif Source	Rank	E-value	Positional Distribution	Matches per Sequence	Similar Known Motifs	Sites

Motif Logo	Motif Source	Rank	E-value	Positional Distribution	Matches per Sequence	Similar Known Motifs	Sites
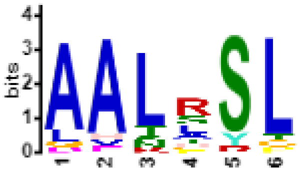	6-AALRSL (STREME)	undefined	1.30e-002			ADP_GLC_PYROPHOSPH_1 (PS00808)	Motif Sites in GFF3

Motif Logo	Motif Source	Rank	E-value	Positional Distribution	Matches per Sequence	Similar Known Motifs	Sites
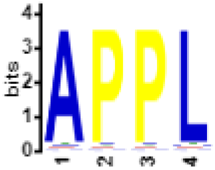	7-APPL (STREME)	undefined	3.54e-002				Motif Sites in GFF3

Motif Logo	Motif Source	Rank	E-value	Positional Distribution	Matches per Sequence	Similar Known Motifs	Sites
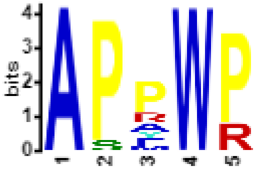	8-APPWP (STREME)	undefined	2.35e-001			VINCULIN_2 (PS00664)	Motif Sites in GFF3

Motif Logo	Motif Source	Rank	E-value	Positional Distribution	Matches per Sequence	Similar Known Motifs	Sites
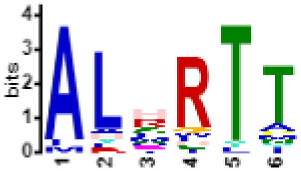	9-ALHRTT (STREME)	undefined	3.12e-001			MOTA (PS01307)	Motif Sites in GFF3

Motif Logo	Motif Source	Rank	E-value	Positional Distribution	Matches per Sequence	Similar Known Motifs	Sites

Motif Logo	Motif Source	Rank	E-value	Positional Distribution	Matches per Sequence	Similar Known Motifs	Sites
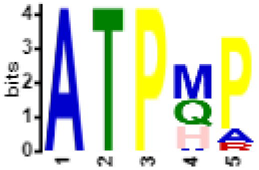	10-ATPMP (STREME)	undefined	3.89e-001				Motif Sites in GFF3

Motif Logo	Motif Source	Rank	E-value	Positional Distribution	Matches per Sequence	Similar Known Motifs	Sites
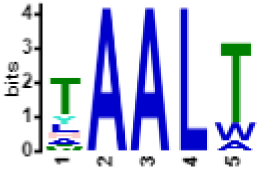	12-TAALT (STREME)	undefined	1.35e+000				Motif Sites in GFF3

Motif Logo	Motif Source	Rank	E-value	Positional Distribution	Matches per Sequence	Similar Known Motifs	Sites
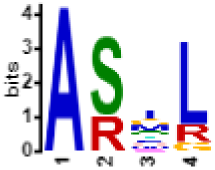	13-ASIL (STREME)	undefined	3.86e+000				Motif Sites in GFF3
