# Supplementary material for: Restriction of the Global IgM Repertoire in Antiphospholipid Syndrome
Source: Front Immunol. 2022 Apr 13;13:865232. doi: 10.3389/fimmu.2022.865232 (PMC9043687; doi:10.3389/fimmu.2022.865232)
Supplement: Supplementary file 16 [file DataSheet_4.pdf]

XSTREME

Motif Discovery and Enrichment Analysis

For further information on how to interpret these results please access https://meme-suite.org/meme/doc/xstreme-output-format.html.
To get a copy of the MEME software please access https://meme-suite.org.

If you use XSTREME in your research, please cite the following paper:
Charles E. Grant and Timothy L. Bailey, "XSTREME: comprehensive motif analysis of biological sequence datasets", *BioRxiv*, 2021.

Motifs | Programs | Input Files | Program information | Summary in TSV Format | Non-redundant Motifs in MEME Text Format

Description

Motifs of mimotopes under-expressed in APS.

Motifs

Enriched motifs (E-value ≤ 0.5 and 3 best STREME motifs).

Expand All Clusters   Collapse All Clusters

Motif Logo	Motif Source	Rank	E-value	Positional Distribution	Matches per Sequence	Similar Known Motifs	Sites
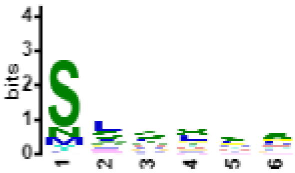	1-SLSSSS (STREME)	undefined	8.93e-895				Motif Sites in GFF3

Show 1 More ↧

Motif Logo	Motif Source	Rank	E-value	Positional Distribution	Matches per Sequence	Similar Known Motifs	Sites
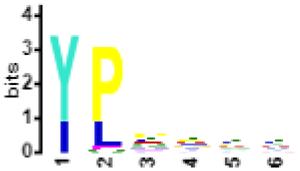	2-YPPTTT (STREME)	undefined	2.73e-073			GLYCOSYL_HYDROL_F2_1 (PS00719) HEXOKINASE_1 (PS00378) MACPF_1 (PS00279)	Motif Sites in GFF3

Motif Logo	Motif Source	Rank	E-value	Positional Distribution	Matches per Sequence	Similar Known Motifs	Sites
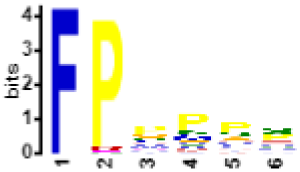	3-FPPPPS (STREME)	undefined	1.66e-031			UPF0012 (PS01227) ALDOLASE_KDPG_KHG_2 (PS00160)	Motif Sites in GFF3

Motif Logo	Motif Source	Rank	E-value	Positional Distribution	Matches per Sequence	Similar Known Motifs	Sites
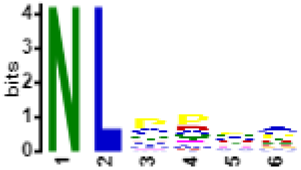	4-NLPPPA (STREME)	undefined	7.85e-020				Motif Sites in GFF3

Motif Logo	Motif Source	Rank	E-value	Positional Distribution	Matches per Sequence	Similar Known Motifs	Sites
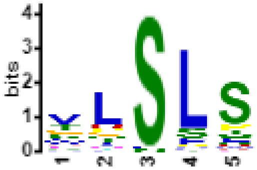	5-VLSLS (STREME)	undefined	1.01e-009			ATPASE_ALPHA_BETA (PS00152)	Motif Sites in GFF3

Motif Logo	Motif Source	Rank	E-value	Positional Distribution	Matches per Sequence	Similar Known Motifs	Sites
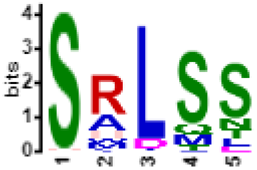	6-SRLSS (STREME)	undefined	7.56e-002				Motif Sites in GFF3

Motif Logo	Motif Source	Rank	E-value	Positional Distribution	Matches per Sequence	Similar Known Motifs	Sites
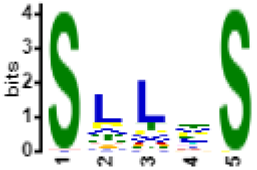	7-SLLTS (STREME)	undefined	1.49e-001				Motif Sites in GFF3

Motif Logo	Motif Source	Rank	E-value	Positional Distribution	Matches per Sequence	Similar Known Motifs	Sites
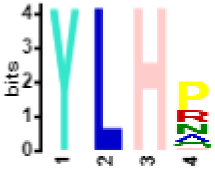	8-YLHP (STREME)	undefined	2.58e-001				Motif Sites in GFF3

Motif Logo	Motif Source	Rank	E-value	Positional Distribution	Matches per Sequence	Similar Known Motifs	Sites
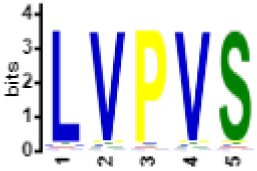	10-LVPVS (STREME)	undefined	6.15e-001				Motif Sites in GFF3

Motif Logo	Motif Source	Rank	E-value	Positional Distribution	Matches per Sequence	Similar Known Motifs	Sites

Motif Logo	Motif Source	Rank	E-value	Positional Distribution	Matches per Sequence	Similar Known Motifs	Sites
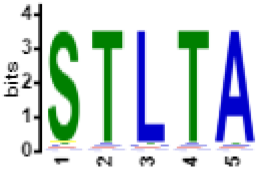	9-STLTA (STREME)	undefined	6.15e-001				Motif Sites in GFF3

Motif Logo	Motif Source	Rank	E-value	Positional Distribution	Matches per Sequence	Similar Known Motifs	Sites
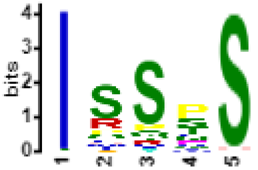	11-ISSPS (STREME)	undefined	1.20e+000			NATRIURETIC_PEPTIDE (PS00263)<br>TUB_2 (PS01201)<br>PR55_2 (PS01025)	Motif Sites in GFF3

Motif Logo	Motif Source	Rank	E-value	Positional Distribution	Matches per Sequence	Similar Known Motifs	Sites
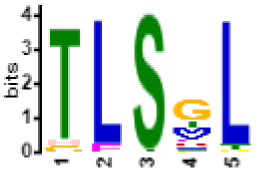	12-TLSGL (STREME)	undefined	1.20e+001				Motif Sites in GFF3
